# Stable establishment of organ polarity occurs several plastochrons before primordium outgrowth in *Arabidopsis*

**DOI:** 10.1242/dev.198820

**Published:** 2021-06-16

**Authors:** Feng Zhao, Jan Traas

**Affiliations:** Laboratoire Reproduction et Développement des Plantes, Université de Lyon 1, ENS-Lyon, INRAE, CNRS, UCBL, 46 Allée d'Italie, 69364 Lyon, France

**Keywords:** Leaf polarity, Sussex signal, Prepattern, Laser ablation, *Arabidopsis*

## Abstract

In many species, leaves are initiated at the flanks of shoot meristems. Subsequent growth usually occurs mainly in the plane of the leaf blade, which leads to the formation of a bifacial leaf with dorsoventral identities. In a classical set of surgical experiments in potato meristems, Sussex provided evidence that dorsoventrality depends on a signal emanating from the meristem center. Although these results could be reproduced in tomato, this concept has been debated. We revisited these experiments in *Arabidopsis*, in which a range of markers are available to target the precise site of ablation. Using specific markers for organ founder cells and dorsoventral identity, we were unable to perturb the polarity of leaves and sepals long before organ outgrowth. Although results in Solanaceae suggested that dorsoventral patterning was unstable during early development, we found that, in *Arabidopsis*, the local information contained within and around the primordium is able to withstand major invasive perturbations, long before polarity is fully established.

## Abstract (precis)

**Summary:** We revisited classical surgical experiments in Solanaceae, using precise laser ablations to show that dorsoventral patterning in vegetative and floral meristems in *Arabidopsis* is robustly programmed in primordia some time before polarity is completely established.

## INTRODUCTION

Plant leaves can adopt a wide range of shapes and sizes depending on the species and the environment in which they develop. In many species, leaves initiate at the flanks of the shoot apical meristem as small bulges. In their most basic form, they then develop as flat, ellipsoid structures, because growth mainly occurs in the plane of the leaf blade. In combination with a restricted expansion in the dorsoventral direction, this leads to the formation of a thin leaf blade with functionally distinct abaxial and adaxial faces, pointing away from and towards the stem, respectively, a property also referred to as leaf polarity.

Several genes have been associated with leaf shape and size, which are determined from the earliest stages of development onwards (e.g. Hervieux et al., 2016; Zhao et al., 2020; Zhu et al., 2020). Key are the regulators involved in establishing leaf polarity and which are activated before or during organ outgrowth. These include, for example: abaxial determinants, such as FILAMENTOUS FLOWER (FIL) (Sawa et al., 1999; Siegfried et al., 1999) or KANADI1 (KAN1) (Kerstetter et al., 2001); adaxial determinants, such as REVOLUTA (REV) or PHABULOSA (PHB) (McConnell et al., 2001); and the so-called margin genes, including *PRESSED FLOWER* (*PRS*) and *WUSCHEL RELATED HOMEOBOX1* (*WOX1*) (Scanlon et al., 1996; Nakata et al., 2012), expressed in the future leaf margin and in the middle domain separating the abaxial and adaxial domains. The network also involves regulation via small RNAs (Kuhlemeier and Timmermans, 2016). Some of the polarity genes are expressed very early, and *REV* and *KAN1* in particular might define a prepattern (Caggiano et al., 2017) even before the upregulation of organ founder cell markers, such as DORNROESCHEN-LIKE (DRNL) (Chandler et al., 2011) or ARABIDOPSIS PHOSPHOTRANSFER PROTEIN 6 (AHP6) (Besnard et al., 2014). If the regulatory network controlling polarity is impaired, leaf and leaf-like organs lose their bilateral symmetry and tend to become axisymmetric (Yamaguchi et al., 2012; Kuhlemeier and Timmermans, 2016).

How is the establishment of polarity coordinated? In a classical set of experiments, Sussex used local incisions in potato meristems to investigate this further (Sussex, 1951; reviewed by Kuhlemeier and Timmermans, 2016). When an incision was made between the vegetative meristem and incipient leaf primordia, leaves did not flatten, but developed into almost axisymmetric organs. This led to the hypothesis that leaf polarity depends on a signal coming from the meristem center. This result was further consolidated in tomato (Reinhardt et al., 2005; Qi et al., 2014; Shi et al., 2017). Reinhardt et al. (2005) showed that ablation in the epidermal L1 layer was sufficient to perturb leaf polarity, suggesting that the signal requires an intact surface layer. Interestingly, this layer is essential in the control of polarized auxin transport during organ formation (Reinhardt et al., 2003; Kierzkowski et al., 2013) and the hormone has also been considered as part of the signal proposed by Sussex. Indeed, certain mutants involved in auxin transport, such as PINOID or PIN-FORMED1, develop axisymmetric lateral organs (Qi et al., 2014) and auxin transport generates differences in hormone concentrations between the periphery and the center of the shoot apical meristem (de Reuille et al., 2006). Qi et al. (2014) proposed that auxin could act as a reverse ‘Sussex signal’, flowing from the adaxial side of the leaf primordium to the adjacent meristem to consolidate leaf dorsoventrality.

However, the concept of a single meristem-centered signal regulating leaf polarity has also been under debate. Although they were able to do so in *Epilobium hirsutum*, Snow and Snow could not confirm the ablation results in potato (Snow and Snow, 1954b). Traditional surgical experiments on meristems mostly rely on the morphology of young leaf primordia as the only marker to define the right position for incision. Therefore, Snow and Snow suggested that cuts leading to a loss in polarity inadvertently caused a reduction in size of the primordia in the radial direction, which then led to abnormal development. More recently, several, more elaborated hypotheses have been brought forward to explain the processes leading to dorsoventrality (Husbands et al., 2009; Caggiano et al., 2017), although all of them require some form of meristem-based signal or information. From a conceptual point of view, Koch and Meinhardt (1994) proposed a model that combined the existence of an organ-inhibiting gradient around the meristem center, with more local, self-organizing patterning processes within the initium itself as soon as the cells leave the inhibiting field. Although this has remained purely hypothetical, findings of Caggiano et al. (2017) in *Arabidopsis* suggest a more complex network of interactions involving auxin transport and pre-existing gradients of several transcription factors, such as KAN1 and REV. The same authors performed a number of laser ablations between the meristem and incipient primordia and showed that the wounds inflicted by cutting can locally induce the expression of certain polarity genes, such as *KAN1*. This result potentially questions the interpretation made by Sussex, because the loss of polarity observed in potato might be due to a wounding reaction involving the ectopic activation of abaxial determinants. However, in *Arabidopsis*, the organs were only followed in small vegetative meristems over short time frames after ablation and no organs with altered polarity were observed. Therefore, we revisited the ablation experiments in *Arabidopsis thaliana*, using very early primordium and polarity markers. We were unable to induce the formation of radialized leaves and sepals by performing ablations along or perpendicular to the radii of vegetative and floral meristems. This was even the case when the ablations were made two or three plastochrons (i.e. the time between successive leaf initiation) before organ outgrowth. Therefore, our results support a model in which organ polarity does not require any meristem-based signal from the earliest stages of primordium specification onwards.

## RESULTS

### Identifying precise targets for ablation using markers for founder cells and organ polarity

To identify the cell populations that would be ablated, we used a set of fluorescent markers for early organ development. For this purpose, we chose *pDRNL:erCERULEAN* as an organ founder cell marker (Chandler et al., 2011). In addition, we used *pFIL:erGFP*. This promoter is active in the abaxial domain of young outgrowing primordia and shows an extended expression in abaxialized mutants (Nakata et al., 2012; Tameshige et al., 2013). We also used *pPRS:SV40-3×GFP* to label the middle domain between both sides of the primordia (Scanlon et al., 1996; Nakata et al., 2012; Xu et al., 2015). Given that the precise relative expression dynamics of these markers has not been described, we constructed a set of lines co-expressing *pDRNL* with either *pFIL* or *pPRS*. To monitor cell growth and lineage, the membrane marker *pUBQ10:Lti6b-tdTomato* was included. In some experiments, propidium iodide (PI) was used for this purpose.

**Fig. 1.**
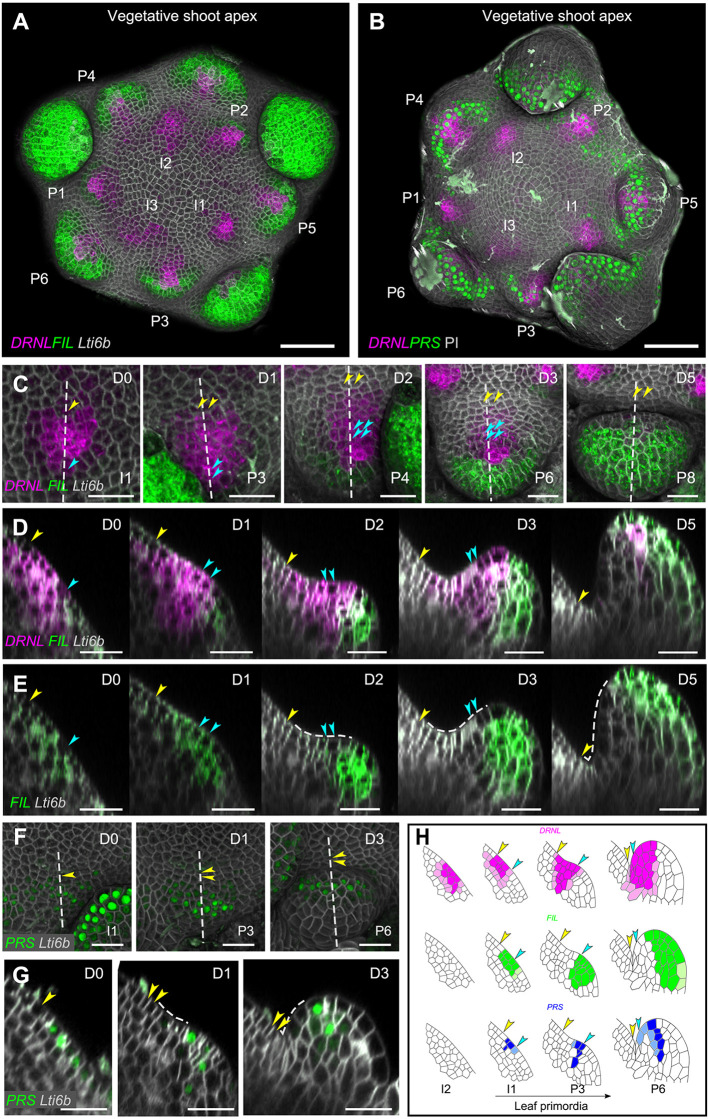
**Dynamic gene expression pattern in vegetative shoot apices of *Arabidopsis*.** (A,B) Top view of 3D reconstruction of vegetative shoot apices co-expressing *pDRNL:erCERULEAN*/*pFIL:erGFP*/*pUBQ10:TdTomato-Lti6b* (A) and *pDRNL:erCERULEAN*/*pPRS:SV40-3×GFP* (B). The stages of leaf primordia are marked according to phyllotactic patterning (from I_3_ to P_6_). The dissected old primordia were trimmed from the projections for clarity. (C-E) Kinetics of *pDRNL* and *pFIL* expression in leaf primordia. The growth of an I_1_ initium was followed for 5 days [Day 0 (D0) to Day 5 (D5)]. (C) Top view of 3D reconstructions. The approximate stages of leaf primordium are shown on the right bottom (I_1_ to P_8_) according to Fig. S1. (D,E) Longitudinal sections of C represented by different fluorescent channels. The sections were taken along the dashed lines indicated in C. Arrowheads mark the cells showing the adaxial (yellow) and abaxial (blue) limits of the initial pDRNL domain and their descendants. *pFIL* expression overlaps with *pDRNL* initially and gradually shifts towards the abaxial side of the leaf primordium. By contrast, *pDRNL* is sustained on the adaxial side of leaf primordium. In P_8_, the *pDRNL* signal is only concentrated at the tips of the leaf primordia. The dashed lines in E indicate the enlargement of the adaxial domain. (F,G) Kinetics of *pPRS* expression in leaf primordia followed for 3 days. (F) Top views of 3D reconstructed images. The approximate primordium stages are indicated (I_1_-P_6_) according to Fig. S1. (G) Longitudinal sections along the dashed lines in F. Yellow arrowheads mark the same cell and its descendants. The adaxial domain is indicated by dashed lines. (H) Schematic of dynamic gene expression patterns in longitudinal sections of leaf primordia at different stages. Arrowheads indicate the cells marking the abaxial (blue) and adaxial (yellow) limits of the inital *pDRNL* domain and their descendants. Scale bars: 20 µm. See also Figs S1 and S2.

In the vegetative meristem, we followed organ initiation from I_3_ (I_1_ being the first incipient primordium that is ready to bulge out, and I_1+_*_n_* the subsequent younger incipient primordia) to P_6_ (P_1_ designates the first visible outgrowing primordium and P_1+n_ the subsequent older outgrowing primordia). In complement to previous results (Chandler et al., 2011), *pDRNL* is active throughout the meristematic dome, with a patchy pattern in the L2 and L3 layers (Fig. S1A,B). However, at the I_3_ position, *pDRNL* activity significantly increases and it also becomes active in the L1 layer (Fig. 1A; Fig. S1A, Fig. S2). Lineage analysis showed that, from I_3_ to I_1_, this activity covers both the future organ boundary and adaxial domain of the future primordium (Fig. 1; Fig. S2) (i.e. a small group of cells, approximately five or six cells in diameter). Slightly later than *pDRNL*-, *pFIL*- and *pPRS*-driven signals become visible at I_1_. They largely overlap with the *pDRNL* maximum, and no apparent polarization of these markers was distinguishable before P_1_, when the primordia start to bulge out. At the P_1_ stage, the activity of *pDRNL* and *pPRS* is more concentrated at the adaxial domain, whereas *pFIL* expression shifts to the abaxial domain and also partially overlaps with the middle domains. Slightly later on, at P_3_, *pPRS* activity becomes restricted to the middle domain only. The polarized expression of the markers is fully resolved when the primordium is clearly growing out and is approximately three cells high (P_4_ in our experimental set-up). Note that *pPRS* signals always extended more towards the lateral organ boundaries compared with the other two markers (Fig. 1; Figs S1, S3).

**Fig. 2.**
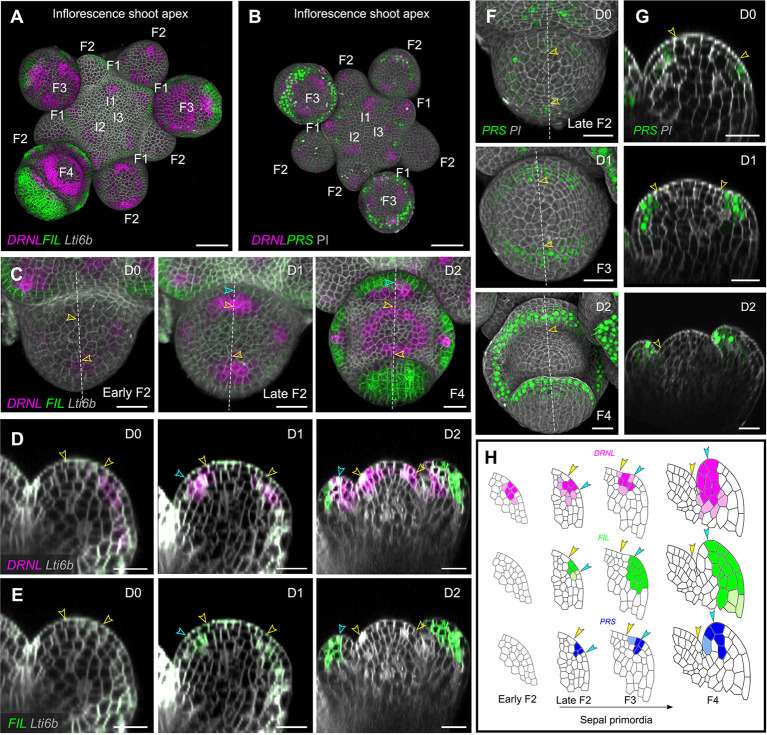
**Dynamic gene expression pattern in inflorescence shoot apices of *Arabidopsis*.** (A,B) Top view of 3D reconstruction of inflorescence shoot apices expressing *pDRNL:erCERULEAN*/*pFIL:erGFP*/*pUBQ10:TdTomato-Lti6b* (A) and *pDRNL:erCERULEAN*/*pPRS:SV40-3×GFP* (B). The successive initia are marked from I_3_ to I_1_; the floral buds (F) are marked at stages 1, 2, 3 and 4. (C-E) Kinetics of *pDRNL* and *pFIL* expression in a floral bud (at early stage 2) followed for 2 days, developing from stage 2 to stage 4. (C) Top view of 3D reconstruction images. (D,E) Longitudinal sections along the dashed line in C showing different fluorescent signals. Arrowheads indicate the cells marking the abaxial (blue) and adaxial (yellow) limits of the inital *pDRNL* domain and their descendants. *pFIL* is activated after *pDRNL*. The initial *pFIL* expression overlaps with that of *pDRNL* and gradually locates at the abaxial side of the sepal primordium. By contrast, the *pDRNL* signals remain on the adaxial side. (F,G) Kinetics of *pPRS* expression in a stage-2 floral bud followed for 2 days. (F) Top views of 3D reconstructions. (G) Longitudinal sections along the dashed line shown in F. Unfilled yellow arrowheads indicate the cell and its descendant. (H) Schematic of dynamic gene expression patterns in sepal primordia from stage 2 to stage 4. Arrowheads indicate the cells marking the abaxial (blue) and adaxial (yellow) limits of the inital *pDRNL* domain and their descendants. Scale bars: 20 µm. See also Fig. S3.

Similar expression patterns of *pDRNL* and *pFIL* were found in the inflorescence meristem: *pDRNL* expression is visible from I_3_ onwards, whereas *pFIL* initiates at I_1_ (Fig. 2A; Heisler et al., 2005; Refahi et al., 2021). During early stage 2 of flower formation, *pDRNL* and *pFIL* expression is restricted to the area where the cryptic bract bulges out slightly, but does not define a clear polarity. Both genes are switched off in the bract during late stage 2 of floral development. Until then, *pPRS* is only activated in a small number of individual nuclei, scattered randomly over the floral bud as well as over the entire inflorescence meristem (Fig. 2; Fig. S3).

Soon after the inactivation of *pDRNL* and *pFIL* in the cryptic bract, the sepal primordia are specified. This is marked by a local activation of *pDRNL*, first in the outer (abaxial) primordium and two lateral primordia (Zhu et al., 2020). At late stage 2, *pDRNL* is activated in all four sepal primordia. Similar to the vegetative meristem, this activation also covers the future boundary with the floral meristem. At floral stages 3 and 4, *pDRNL* activity becomes restricted to the adaxial side of the sepal primordia and the promoter is also activated in a ring-like domain in one of the future inner whorls, probably corresponding to part of the third whorl (Fig. 2A-C). The activation of *pFIL* and *pPRS* is slightly later than that of *pDRNL* but still during late floral stage 2. In the leaf, *pFIL* is initially activated at the sepal primordia in a pattern that overlaps with that of *pDRNL*. At late floral stage 2, only the outer sepal is marked, followed by the inner and lateral sepals (see also Zhu et al., 2020) during stage 3. Similar to the leaf primordia (Fig. 1C-E,H), *pFIL* expression shifts peripherally during stage 3 and becomes limited to the abaxial and middle domains of the sepals at stage 4 (Fig. 2C-E,H; Fig. S3). *pPRS* is first activated in the outer sepal at late stage 2, partially overlapping with the *pDRNL* expression domain. Subsequently, *pPRS* is mainly expressed in the adaxial side of the sepal primordia. Gradually, *pPRS* becomes active in a ring covering the middle domain of all sepals as well as the lateral boundaries between them during stage 4 (Fig. 2B,F-H; Fig. S3).

In summary, although the staging of primordia can vary a little depending on the size of meristems, the relative expression dynamics of the three different markers allows us to identify the precise moments when the organ founder cells and the different domains that characterize organ polarity are established. In this context, the upregulation of *pDRNL* marks the future organ boundary and adaxial domain, whereas, in later stages, *pFIL* marks the abaxial domain and the middle domain, the latter being characterized in addition by *pPRS* expression. We next used these patterns to characterize the precise locations of the ablations. We distinguished two categories: ablations that did not or only marginally touched the expression domains; and ablations within specific domains of the initia or primordia. These will be discussed separately.

### Ablations next to early leaf initia/primordia do not perturb leaf dorsoventrality in *Arabidopsis*

**Fig. 3.**
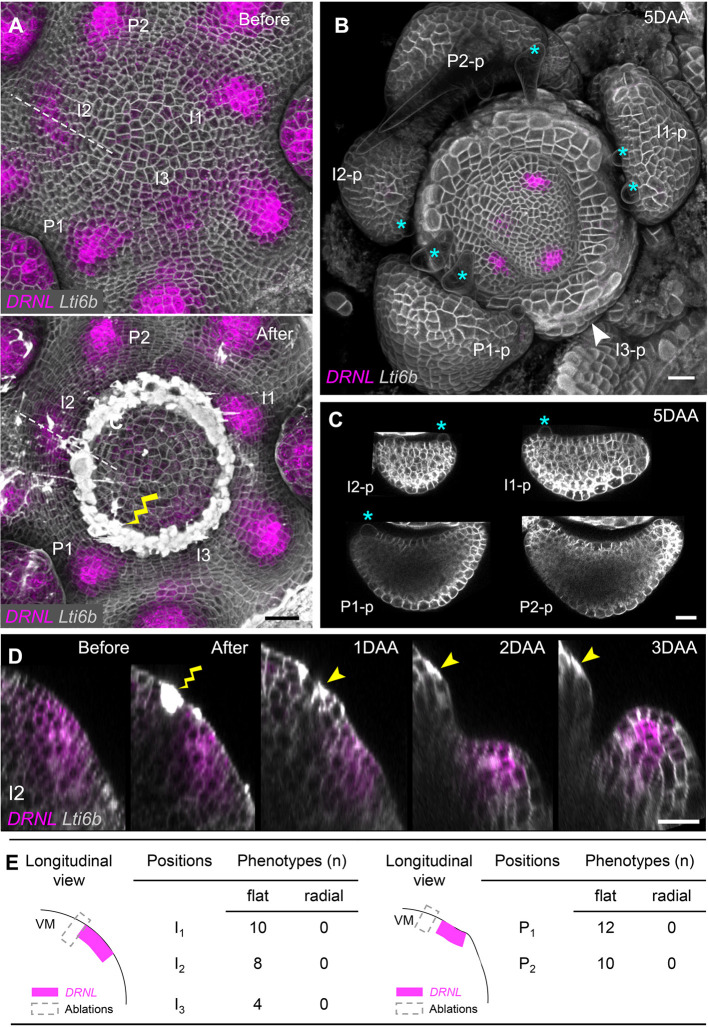
**Circular ablation next to initia or primordia in vegetative meristems do not perturb polarity in *Arabidopsis*.** (A) Top view of 3D reconstruction of vegetative shoot apex expressing *pDRNL:erCERULEAN* and *pUBQ10:TdTomato-Lti6b* before (upper panel) and after (lower panel) laser ablation. The age of leaf primordia is indicated by I_3_-P_2_. The wound caused by ablation is indicated by the yellow lightning sign. (B) Top view of the VM at 5 DAA. The growth of leaf primordia shown in A was followed and marked as I_3_-p to P_2_-p referring to the original I_3_-P_2_ positions. A distal part of I_3_ was ablated, after which only limited outgrowth in the form of a ring formed (indicated by the white arrowhead) and *pDRNL* activity was lost. (C) Cross-sections of I_2_-p to P_2_-p in B, showing the flattening of these leaves. The adaxial cell fate is characterized by the formation of trichomes, indicated by blue asterisks in B and C. (D) Growth dynamics of the I_2_ initium during 3 days after ablation, represented by longitudinal sections along the dashed lines in A and B. The wound is marked by a yellow lightning sign and yellow arrowheads. The circular ablation did not change the expression pattern of *pDRNL*. (E) Summary of the wounding position and corresponding phenotypes after circular ablation next to individual initia/primordia in VMs 5 DAA. Scale bars: 20 µm. See also Fig. S4 and S5. DAA, days after ablation; VM, vegetative meristem.

We first sought to repeat experiments in which young primordia were physically separated from the central meristem through incision, which, in potato and tomato, resulted in the formation of abaxialized, axisymmetric organs. To this end, we performed circular ablations surrounding the meristem center (i.e. perpendicular to the meristem radius) using *pDRNL* as an early leaf initium marker (Fig. 3A). To exclude short-distance signal transmission between the meristem and the young leaves, the incisions were set as close as possible to the incipient primordia. In 12 meristems, 22 I_3_-I_1_ primordia were left intact or only a few proximal cells were eliminated. In addition, we were able to follow 22 intact P_1_-P_2_ primordia in these meristems. Under these conditions, none of the leaves that grew out showed radial symmetry (Fig. 3; Figs S4, S5, Fig. S6A,B). Polarized leaves grew out even following ablations performed at I_3_, when *pDRNL* upregulation was only starting and *pFIL* was not expressed at all (Fig. 1A; Fig. S1C). However, we could not determine whether there was any transient change in polarity. Nevertheless, the patterning process did not require a meristem-based signal.

In tomato, not only incisions isolating the primordium from the central meristem cause a loss of dorsoventrality. In addition, incisions parallel to the meristem radius (i.e. at the lateral boundaries between organs) are sufficient to induce abaxialized organs (Shi et al., 2017).

**Fig. 4.**
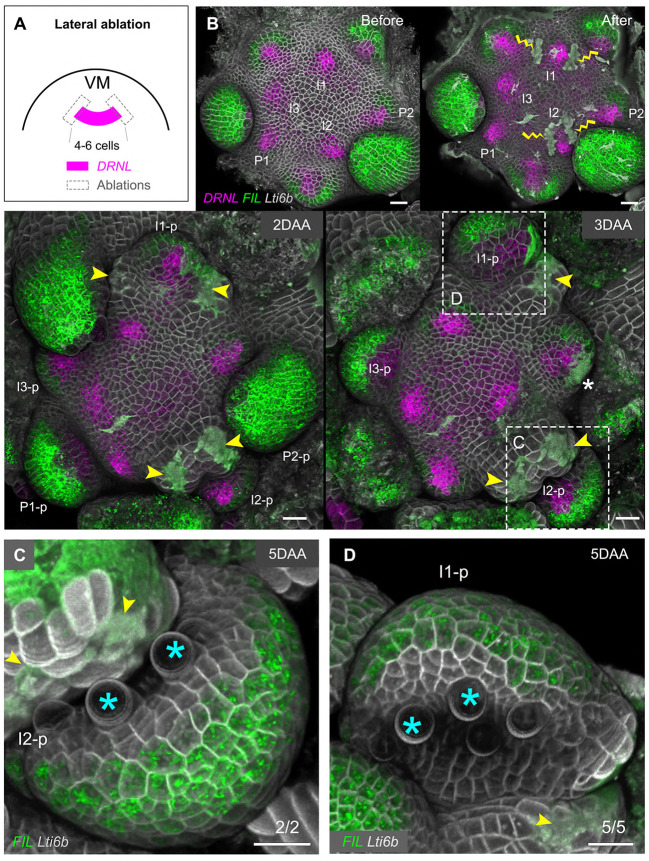
**Lateral ablation in vegetative meristems next to or marginally touching the initia and primordia do not perturb polarity in *Arabidopsis*.** (A) Schematic of the lateral ablations in VM. (B) Overview of representative VM before and after laser ablation. The growth of the meristem was followed for 5 days. The wounds are marked by yellow lightning signs and yellow arrowheads. The initial stages of leaf primordia before or after ablation are indicated by I_3_-P_2_, their respective later stages as I_3_-p to P_2_-p as in Fig. 3 in the main text. The wound marked by a white asterisk on 3 DAA was caused by dissecting away P_2_-p at 2 DAA. (C,D) Detailed shapes of I_2_-p and I_1_-p (boxed areas in B) at 5 DAA. The trichomes initiated from the adaxial leaf surface are marked by blue asterisks. Yellow arrowheads indicate the wounding sites. Scale bars: 20 µm. DAA, days after ablation; VM, vegetative meristem.

In two I_2_ and five I_1_ initia, the ablations were four-to-six cells apart and either left the initium (five or six cells wide) intact or only slightly reduced its width. In all seven cases, the resulting primordia showed a polarized distribution of *pDRNL* and *pFIL* signals 2 or 3 days after incision (Fig. 4A,B). Finally, a characteristic leaf with dorsoventral identity formed 5 days after incision (Fig. 4C,D). Once the primordia had already started to bulge out, lateral ablations of three-to-eight cells wide did not affect growth and leaf polarity (13/13 of P_1_ and P_2_ primordia examined) (Fig. S7).

Within the limits of the markers that we used, we did not find any indication that lateral ablations at the boundaries and ablations between the vegetative meristem and initiating organs perturbed polarity from I_3_ onwards (i.e. before *pFIL* and *pPRS* are activated).

### Laser ablations next to early sepal initia in the floral meristem do not perturb dorsoventrality in *Arabidopsis*

**Fig. 5.**
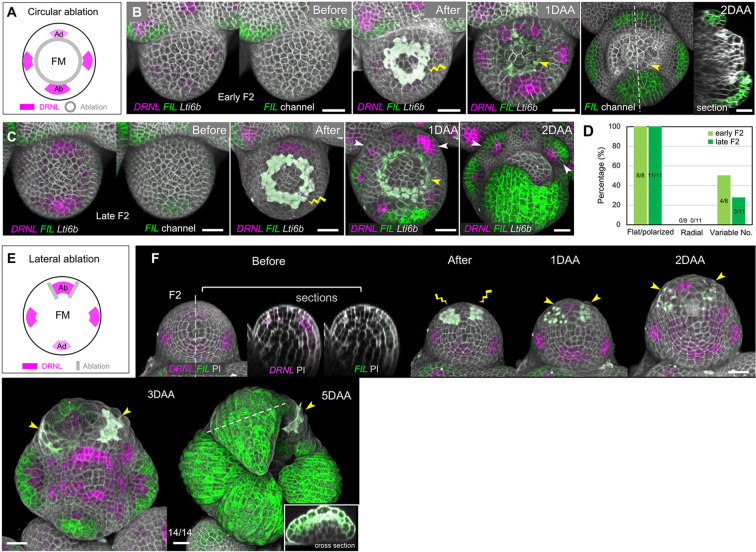
**Ablations in floral meristems in *Arabidopsis*.** (A) Schematic of the circular ablation strategy in FM. The outer (abaxial) and inner (adaxial) sepal initia are marked as Ab and Ad, respectively. (B) Representative example of circular ablation in an early stage-2 floral bud (Early F2). The growth of the floral bud was followed for 2 days. *pFIL* was not yet activated before the ablation. The wounds are marked by a yellow lightning sign and yellow arrowheads. (C) Representative example of circular ablation in a late stage-2 floral bud (Late F2), when the *pFIL* activation begins. White arrowheads indicate the ectopically initiated sepal primordia. In all cases in B and C, circular ablation did not affect the sepal polarity. (D) Quantitative summary of B and C in terms of the percentages of sepal shapes and polarity. Numbers indicate the number of floral buds in a particular category out of the total number (8 early and 11 late F2) observed. (E) Schematic of the lateral ablation strategy in FM. The outer (Ab) sepal primordia in stage-2 floral buds were confined by two lateral incisions. (F) Representative example of lateral ablations in a stage-2 floral bud. At this stage, the *pFIL* activity was barely visible before the ablation. The wounds are marked by yellow lightning signs and yellow arrowheads. After the ablations, the growth was followed for 5 days and polarity was not compromised in any of the 14 primordia. Inset shows a cross-section marked by the dashed line in the main image. Scale bars: 20 µm. DAA, days after ablation; FM, floral meristem.

We next assessed the effect of laser ablation on sepal shape and polarity at the floral meristem. As performed for the vegetative meristem, circular ablations were used to isolate sepal initia from the central meristem. In 7/19 stage-2 floral buds, this led to an increase in sepal number, the remaining 12 buds had four sepals, as in wild type (WT) (Fig. 5A-D). None of the sepals was radially symmetric. This was even the case when early stage-2 flowers were ablated (i.e. before *pFIL* expression was activated) (8/8 meristems, Fig. 5A,B,D).

We then confined sepal initia by two parallel ablations at the lateral boundaries in stage-2 floral buds. All 14 sepal initia established normal polarity and no radial sepals were formed (Fig. 5E,F).

In summary, in the floral meristems, neither parallel nor perpendicular ablations close to the initia perturbed dorsoventrality.

### Ablations within the leaf initia/primordia either are followed by regeneration of dorsoventral organs or lead to growth arrest

As mentioned earlier, Snow and Snow (1954a) proposed that the incisions made by Sussex could have led to a reduction in size of the initia, thus causing the formation of radialized leaves. We further sought to confirm this with a set of ablations in which either the adaxial or abaxial domain of the initia/primordia was eliminated or initium/primordium width was reduced to only one to four cells.

#### Adaxial ablations

**Fig. 6.**
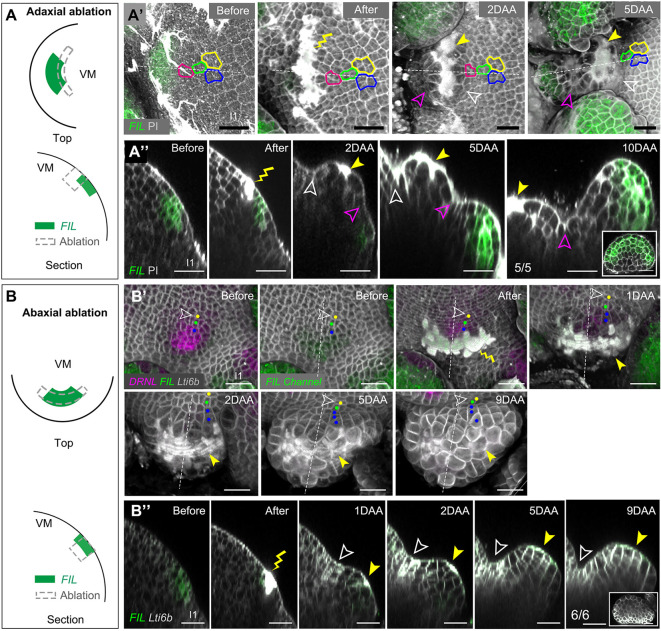
**Ablations parallel to meristem circumference, within leaf initia/primordia of *Arabidopsis*.** (A-A″) Ablations of the adaxial part of the *pFIL* expression domain before *pFIL* polarizes. (A) Schematic of the wounding position from a top (upper panel) and longitudinal section view (lower panel). (A′) Top view of a vegetative meristem showing an example of I_1_ leaf initium before and up to 5 days after ablation. The precise wounding sites are marked by either yellow lightning signs or yellow arrowheads. Cell lineages are marked by different colors (yellow, blue, green or pink outlines). (A″) Longitudinal sections of the same primordium along the dashed lines in A′. At 2 DAA, *pFIL* activity is substantially reduced. The initial organ boundary starts to fold (unfilled white arrowheads), while a bulge starts to grow out, and an additional boundary is defined (unfilled magenta arrowhead). After 10 days, a polarized leaf with abaxial *pFIL* labeling is formed. Cross-section of the leaf is shown in the inset. The same results were obtained from five different I_1_ or P_1_. (B-B″) Ablations including most of the abaxial *pFIL/pDRNL* expression domain. (B) Schematic of the wounding position from a top (upper panel) and longitudinal section (lower panel). (B′) Outgrowth after ablation at I_1_ position. A small outgrowth is formed after 9 days. Colored dots show the different cell lineages. (B″) Longitudinal sections along the dashed lines in the same sample as in B showing that *pFIL* activity is lost in the outgrowth. The inset shows the transverse sections of the outgrowth at 9 DAA. The wounding sites are marked by a yellow lightning sign or yellow arrowheads. Unfilled white arrowheads indicate the organ boundary. The same results were obtained from six I_1_ or P_1_. Scale bars: 20 µm. DAA, days after ablation.

We also performed more restricted ablations, limited to an arc of the circumference, targeting single initia. These were sufficiently large to ablate up to the entire adaxial half of the *pFIL* expression domain. After ablation at I_1_ or P_1_, polarized leaves were formed (*n*=5; Fig. 6A-A″; Fig. S6C). However, this was correlated with an initial inactivation of *pFIL*, followed by a more distal reactivation of *pFIL* toward the meristem periphery and adaxial cell regeneration from the abaxial side of the wound (Fig. 6A-A″). Notably, this also involved a redefinition of the organ boundary with the meristem (Fig. 6A′,A″).

#### Abaxial ablations

Twelve of the circular ablations described above went through I_3_-I_1_ initia, eliminating the abaxial part of the *pDRNL*-labeled domain (see Fig. S5 I_3_ for an example of precise positions). In all cases, growth arrest was observed. Some outgrowths occurred next to the wound, but these did not develop further and did not express *pDRNL* (Fig. 3A,B; Fig. S4). We also performed six more restricted ablations, limited to an arc of the circumference, targeting single initia. This resulted in an elimination of 60-80% of the entire *pFIL* domain, leaving only a few cells adaxially. In these cases, the outgrowth of leaf primordia was also halted, showing limited cell division of the remaining adaxial cells and loss of *pFIL* expression (Fig. 6B′,B″; Fig. S6C).

#### Lateral ablations

**Fig. 7.**
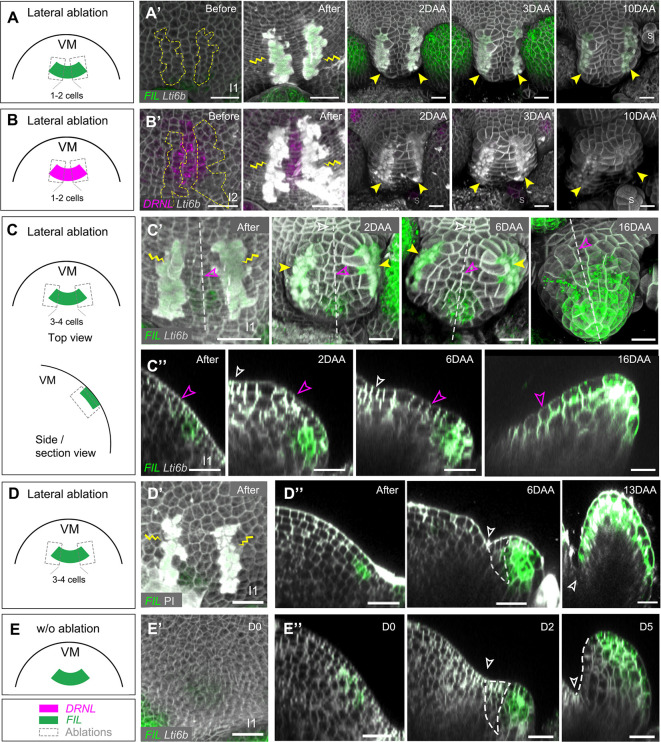
**Lateral ablations within leaf initia of *Arabidopsis*.** (A-B′) Lateral ablations leaving a row of approximately one to two initium cells intact led to the inhibition of leaf outgrowth in I_1_ (A,A′) and I_2_ (B,B′) and loss of *pFIL* and *pDRNL* expression. (A,B) Schematic showing the positions of the ablations. (A′,B′) Top views of representative ablations. The same results were obtained from two I_1_ and two I_2_ leaf initia. The contours of the corresponding ablation zones are indicated in the left panel (before ablation) of A′ and B′. ‘S’ in the right panels indicates the newly initiated stipules. (C-C″) Representative example of lateral ablation (three or four cells apart) at I_1_ showing reduced growth from 0 to 16 DAA. (C) Schematic showing the wounding positions. (C′) Top view. (C″) Longitudinal sections along the dashed lines shown in C′. Unfilled magenta arrowheads mark the same cell wall over time. Polarity is not perturbed up to 6 DAA, but then leads to the formation of a small abaxialized structure 16 DAA, with *pFIL* activity extending adaxially beyond the unfilled magenta arrowhead. (D-D″) Another example of a lateral ablation (three or four cells apart) at I_1_. (D) Schematic showing the wounding position. (D′) Top view. (D″) Longitudinal sections showing leaf growth for 13 days. Unfilled white arrowheads show the organ boundaries. Dashed white lines indicate the adaxial domains. (E-E″) The development of a control leaf without wounding. (E) Schematic of the leaf initia in the intact meristem. (E′) Top view of a typical I_1_ leaf initium. (E″) Longitudinal sections showing the leaf growth for 5 days. Dashed white lines indicate the adaxial domains. Unfilled white arrowheads show the organ boundary. Yellow lightning signs and yellow arrowheads mark the wounding sites. Scale bars: 20 µm. See also Fig. S7. DAA, days after ablation.

As we have discussed, lateral ablations leaving most of the leaf primordia or initia intact did not affect development. We next analyzed a set of lateral ablations at an early stage of primordia development, eliminating a significant part of the founder cells. In four cases (at two I_1_ and two I_2_), the incisions were very close (i.e. one or two cells apart). These initia did not further develop and the signal of *pDRNL* or *pFIL* was lost (Fig. 7A-B′). Thirteen ablations at I_1_/I_2_ were three or four cells apart. Seven of the resulting primordia then developed normally (Fig. S6C). However, in six cases, growth was dramatically reduced, leading to the formation of small bulges. Polarity in these primordia was maintained for at least 6 days. Later on, the abaxial *pFIL* marker extended adaxially (Fig. 7C-E″) in these small, non-growing primordia. This apparent loss of polarity could be due to indirect effects, possibly related to their limited growth.

### Ablations within the sepal initia do not perturb organ polarity

**Fig. 8.**
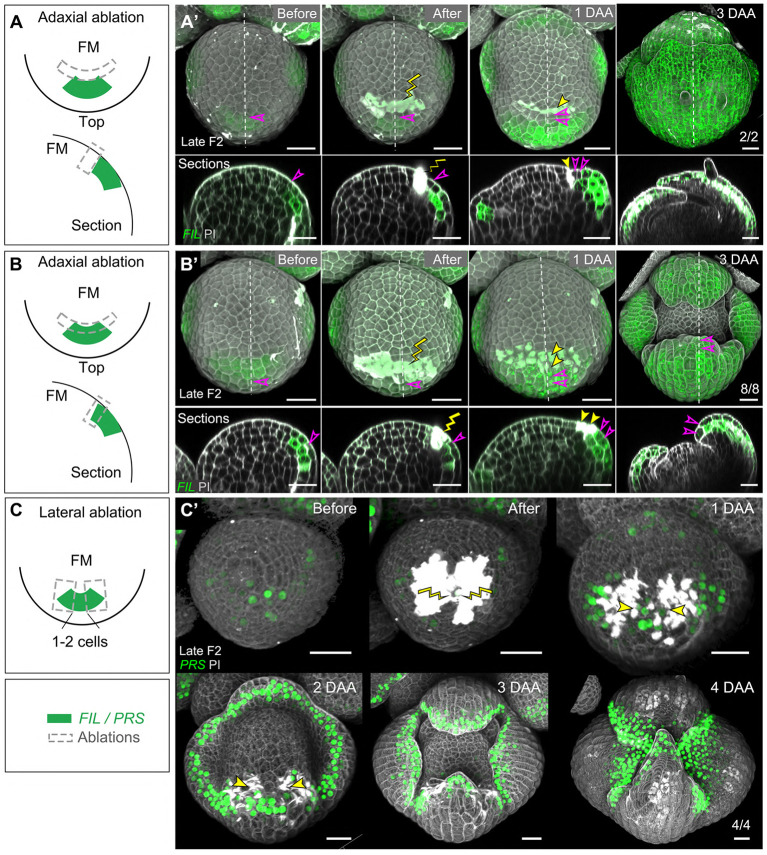
**Ablations parallel to the meristem circumference, within sepal initia of *Arabidopsis*.** (A,A′) Ablation next to the *pFIL* domain. A shows a schematic and A′ a typical example of normal sepal development. Upper panels show the top views and the lower panels the longitudinal sections along the dotted lines. (B,B′) Ablation eliminating the adaxial part of the *pFIL*-labeled domain. B shows a schematic and B′ shows a typical example of sepal development showing normal polarity. Upper panels show the top views and lower panels the longitudinal sections along the dotted lines. (C,C′) Lateral ablation in a stage-2 floral bud expressing the *pPRS:SV40-3×GFP* marker. (C) Schematic showing the wounding position. (C′) A typical example of sepal development, in which only a cell was left in between the wounds. Growth was followed for 4 days and a flat sepal was formed. Yellow lightning signs and yellow arrowheads mark the wounding sites. Unfilled magenta arrowheads mark the cell next to the wound and its descendants. Scale bars: 20 µm. DAA, days after ablation.

We next performed ablations on the floral meristem, restricted to an arc of the circumference, eliminating part of the sepal before it started to grow out. As a control, restricted ablations next to the initium did not perturb development, as was also observed for circular ablations (*n*=2, Fig. 8A-A′; compare Fig. 5A-D). This was also the case when the adaxial part of the *pFIL* domain itself was killed (*n*=8, Fig. 8B-B′).

When we performed lateral ablations eliminating part of the initia themselves and reducing their width, we could not observe any effect on polarity. Notably, even leaving one radial row of initium cells did not perturb sepal polarity, although the sepals were narrower (*n*=4, Fig. 8C-C′).

We finally eliminated all founder cells of the outer sepal primordia. In 3/3 successful ablations, no sepal regenerated along the dorsal-ventral axis. However, to our surprise, flat sepals were re-initiated from the lateral boundaries, which was characterized by the ectopic activation of *pDRNL* in these domains (Fig. S8A). To test whether the reactivation of *pDRNL* is induced by signals from the neighboring sepal primordia, we eliminated the founder cells of the outer and lateral sepal primordia in five floral buds. In all cases, *pDRNL* was activated in the lateral boundaries and new flat sepals developed (Fig. S8B). As shown above, the margin promoter *pPRS* is active in these lateral boundaries (Fig. 2). *PRS* has a role in cell proliferation and is important for the lateral extension of sepals (e.g. Zhao et al., 2020). Therefore, we investigated whether sepals would also regenerate from boundaries in mutants with a reduced margin activity. This was not the case, and no regeneration was observed from the boundary when the outer and lateral sepal primordia in *wox1 prs* double mutants were ablated (*n*=5, Fig. S8C).

## DISCUSSION

**Fig. 9.**
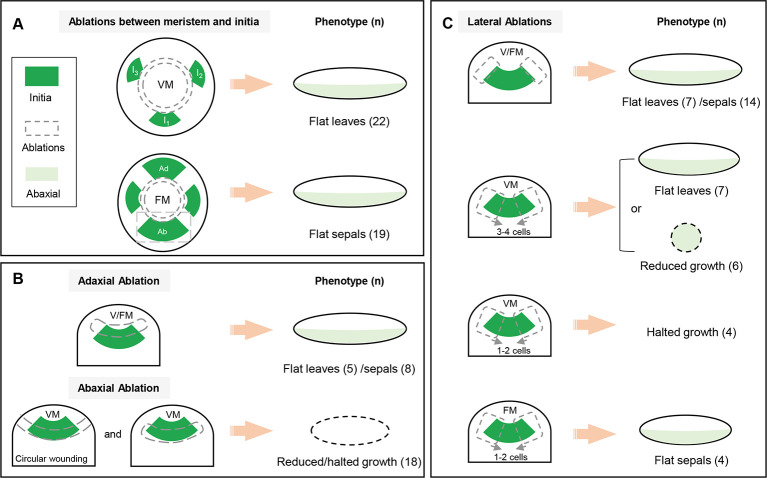
**Schematics** **of laser ablations** **in *Arabidopsis***. (A) A number of circular ablations did not touch, or only marginally touched, the leaf initia (at VM) and sepals (at FM). These systematically developed into flat shapes with normal polarity. (B) The killing of adaxial parts of young initia did not perturb organ polarity in VM and FM (upper panel). The wounds in the abaxial part of young initia caused reduced or halted organ growth in VM (lower panel). (C) Lateral ablations that left a large area of initia/primordia intact did not perturb organ flatness and polarity in VM and FM (upper panel). When the wounds were kept three or four cells apart in the leaf initia, the leaves became flat or showed a dramatically reduced final size, which could be abaxialized after longer periods. The leaf initia stopped growing when the wounds are only one or two cells apart in the VM (middle panels). However, even if only one or two sepal initia cells were left after wounding, flat sepals were still able to grow out (lower panel). FM, floral meristem; VM, vegetative meristem.

In this study, we combined *in vivo* imaging with laser ablation to study the establishment of organ polarity in both vegetative and floral meristems (for a summary of the different types of ablations, see Fig. 9A-C). In particular, the use of larger vegetative meristems grown in short days allowed us to monitor leaf development over long periods. This is an important advantage over previous work, in which more precise lineage tracking was not possible.

We found that organ polarity does not require any meristem-based signal at least two or three plastochrons before the organs grow out, at a point when *pDRNL* is upregulated and organ founder cells are selected. Likewise, sepal polarity becomes autonomous at stage 2 of flower development (i.e. when sepals are initiated, but have not yet started to grow) (Fig. 9A).

Husbands et al. (2009) proposed that leaf polarity is determined in three phases: establishment, resolution and maintenance. The main players would be activated during the first phase, but not necessarily in the correct pattern, which would be defined during the second phase and maintained thereafter. Although the first two phases would, in principle, require external information, local molecular regulation within and around the leaf primordium itself should be sufficient for maintenance once it is fully deployed. Although results in tomato and potato suggested that the dorsoventral patterning was not very robust during the initiation process itself (Sussex, 1951; Reinhardt et al., 2005), we show here that, even at a stage when major regulators, such as *FIL* or *PRS*, are not yet activated, polarity is already robustly established and maintained. Indeed, although the ablations cause major changes in local signaling and even can affect gene expression (Heisler et al., 2010; Caggiano et al., 2017; Zhao et al., 2019), we did not see any effect on leaf polarity, suggesting that an external signaling gradient does not have a role once *pDRNL* is activated. Further work is now required using additional markers to show that there are no more-subtle effects on ad/abaxial polarity.

To understand this early establishment of polarity, the results reported by Caggiano et al. (2017) are particularly relevant. They showed the existence of concentric expression zones around the meristem center of genes involved in organ polarity, a domain weakly expressing the adaxial determinant *REV* in the meristem center, surrounded by cells expressing the abaxial gene *KAN1* at the meristem periphery. Both transcription factors mutually inhibit each other and the boundary between the two domains covers cells that have not yet been incorporated in new primordia (i.e. this boundary precedes organ formation). Therefore, both genes appear to define some type of ‘prepattern’ for polarity. At sites where auxin accumulates, *REV* is further upregulated, at least from I_2_ onwards (Caggiano et al., 2017). Our data suggest that this upregulation is also accompanied by increased *DRNL* expression, and there is evidence that both transcription factors directly interact (Chandler et al., 2007; Zhang et al., 2018). Therefore, we deduce that, initially, both REV and DRNL cover the future organ boundary and the adaxial domain. At a later stage, when tissue folding starts at the boundary, auxin levels decrease. Combining our results with those of Caggiano et al. (2017), we propose that this is followed by the inactivation of both *REV* and *DRNL* and the activation of *KAN1* in the forming organ boundary.

Caggiano et al. (2017) also suggested that the loss of polarity observed by Sussex (1951) after incision between the meristem and the young primordia could be the result of perturbation of this prepattern as a wound reaction. As we have shown here, ablations substantially modify the meristem structure, causing local outgrowth. In addition, it has been shown that ablation can alter auxin fluxes, microtubule organization and gene expression patterns (Heisler et al., 2010; Caggiano et al., 2017; Zhao et al., 2019). In particular, the polarity gene *KAN1* is induced by wounding. Caggiano et al. (2017) suggested that this ectopic expression might create a zone of abaxial identity and organs formed in this zone are then abaxialized. This might explain the loss of polarity observed in tomato and potato. We did not obtain any clear evidence for this scenario, because we never obtained abaxialized leaves or sepals after ablation. However, we did observe changes in local patterning. When a large part of the future adaxial domain was eliminated, *pFIL* activity was initially lost, a new boundary was respecified, and a polarized primordium was formed a short distance from the wound after ablation (Fig. 6, Fig. 9B). This could indicate that the ablation leads to some type of dedifferentiation followed by local repatterning. No meristem-based signal would be required for this process, because the initium was cut off by the ablation. It is possible that ectopically induced KAN1 expression would be sufficient to guide such a reprogramming.

Nevertheless, the results from *Arabidopsis* are very different from those reported for Solanaceae. A possible explanation for this discrepancy is that the expression dynamics and roles of polarity determinants differ between species. For example, this is illustrated by small RNAs targeting auxin response factors (ARFs), which are implicated instead in the maintenance phase in the developing leaves of *Arabidopsis*, whereas they are indispensable for the establishment of polarity in several monocot species [see Kuhlemeier and Timmermans (2016) for discussion].

A final issue concerns the lateral organ boundaries (Fig. 9C). Working on tomato, Shi et al. (2017) found that lateral ablations at the level of organ boundaries are already sufficient to perturb polarity, suggesting a role of these boundaries in organ formation. Although we found changes in *pFIL* expression after some of the lateral ablations, these changes were always correlated with the compromised outgrowth of leaves. The *FIL* expression domain was reduced in width and even a loss of *pFIL* signals was observed in cases where very few cells were left (Fig. 7A,A′). In these cases, we could not unambiguously uncouple growth arrest and the apparent loss of polarity, because both processes occurred simultaneously. When the ablations were slightly further apart, some growth occurred and *pFIL* could also extend adaxially. However, this happened long after growth arrest and could be largely indirect. The link between growth and FIL has remained unclear. Eshed et al. (2004) and Goldshmidt et al. (2008) suggest that FIL could be growth promoting, whereas Refahi et al. (2021) did not find a clear correlation between growth rates and *FIL* expression in the *Arabidopsis* flower.

Interestingly, ablations of the entire sepal in the *Arabidopsis* flower affected boundary zones outside the domain of *pDRNL* expression, but where the margin promoter *pPRS* was still expressed (Fig. 2B). We have previously shown that margin genes are involved in extending the outgrowing sepal primordium as an important step in maintaining organ flatness (Zhao et al., 2020). We show here that the lateral boundary cells initiate new sepals precisely when the entire organ is ablated. This regeneration process requires PRS, suggesting that, under normal conditions, the gene maintains the competence of the lateral boundary cells to respecify into a polarized sepal.

In conclusion, we find that, in *Arabidopsis*, the local information within and around the primordium is able to withstand major perturbations in local shape and signaling, long before polarity is fully resolved. In this study, we used three different markers involved in organ initiation and polarity. Extension of this approach to other polarity markers should provide further insight into how the molecular regulatory network functions and reacts to perturbation.

## MATERIALS AND METHODS

### Plant material and growth conditions

The mutant [*wox1 prs* (Nakata et al., 2012)] and marker lines [*pFIL:erGFP* (Watanabe and Okada, 2003), *pDRNL:erGFP* (Cole et al., 2009), *pDRNL:erCERULEAN* (Cole et al., 2013), *pPRS:SV40-3×GFP* (Xu et al., 2015) and *pUBQ10:Lti6b-tdTomato* (Segonzac et al., 2012)] used in this study have been previously described. The double or triple fluorescent labeling marker lines were obtained by crossing and subsequently confirmed by antibiotic screening and checking of the fluorescence signals. To obtain vegetative shoot apical meristems, plants were grown under short-day conditions on soil (8 h light/16 h dark period) for 33-37 days until further dissections. To obtain inflorescence meristems, plants were first cultured in short-day conditions for 3 weeks and then transferred to long-day conditions (16 h light/8 h dark period) for an additional 2 weeks. Under these conditions, the shoot apical meristems were big enough to facilitate both dissection and laser ablations. Plants grown on soil were cultured with 60% humidity, using light-emitting diodes (150 mEm^−2^s^−1^) and at a daytime temperature of 20-22°C. For live imaging and long-term culture, the meristems were dissected and cultured *in vitro* on apex culture medium (ACM) (Hamant et al., 2019) supplemented with 200 nM N6-benzyladenine and 0.1% plant preservative mixture (PPM; Plant Cell Tech).

### Sample preparation

To prepare the vegetative meristems, 33- to 37-day-old rosettes were carefully removed from the soil to preserve the root as much as possible. The rosettes were then washed with water and dried with tissue paper. Whole plants were moved to dissection plates (3% agarose in Petri dishes) and immobilized as previously described (Shi et al., 2020). To expose the vegetative meristem, the old leaves were cut off as cleanly as possible using injection needles (0.45 mm, BD). The young leaf primordia covering the meristem were dissected away carefully using tweezers under a binocular microscope (Leica M125) at high magnification. To prepare inflorescence meristems, whole-shoot apices were inserted in the ACM and dissected with tweezers. After dissection, the inflorescence shoot apices were transferred to a new ACM plate and cultured *in vitro* until further experiments. We usually left the dissected vegetative and inflorescence meristems to recover for ∼4 h before imaging and ablations.

### Laser ablations

The laser ablation experiments were performed as described by Zhao et al. (2019), using a Zeiss LSM 700 laser-scanning confocal microscope, equipped with an Andor MicroPoint laser ablation system driven by iQ3 software (Oxford Instruments), which delivered a 6-Hz paused laser at 356 nm. Pre-staining of the meristems with PI (Sigma-Aldrich, 100 mM) for 5 min was carried out to increase the efficiency of laser ablation. To determine the precise site of ablation, we first made an acquisition using Zeiss ZEN software (black edition). We then adjusted the focal plane and chose the circular or freeline tool in iQ3 to draw the trajectory for the ablation according to the position shown in the confocal image. We set the laser power at level eight or nine, with five repetitions for each point on the trajectory in order to make a deep wound. After the ablation, we made another acquisition using the confocal microscope. Under such conditions, our ablations were usually approximately two or three cells deep. In total, 60 ablations in vegetative meristems were carried out: 38 were two or three cells deep, 19 were three or four cells deep, and three were one or two cells deep.

### Live imaging and image processing

For live imaging, the dissected meristems were immersed in distilled water supplemented with 0.1% PPM and examined in a Zeiss LSM 700 laser-scanning confocal microscope equipped with water immersion objectives (W Plan-Apochromat 40×/1.0 differential interference contrast). For three-dimensional (3D) image processing, we used the Zeiss ZEN software (black edition), choosing the transparency rendering mode in order to visualize the signals on the meristem/organ primordia surface. Longitudinal optical sections of the stacks were carried out using Fiji freeware.

## Supplementary Material

Supplementary information

Reviewer comments

## References

[DEV198820C1] Besnard, F., Refahi, Y., Morin, V., Marteaux, B., Brunoud, G., Chambrier, P., Rozier, F., Mirabet, V., Legrand, J., Lainé, S. et al. (2014). Cytokinin signalling inhibitory fields provide robustness to phyllotaxis. *Nature* 505, 417-421. 10.1038/nature12791 24336201

[DEV198820C2] Caggiano, M. P., Yu, X., Bhatia, N., Larsson, A., Ram, H., Ohno, C. K., Sappl, P., Meyerowitz, E. M., Jönsson, H. and Heisler, M. G. (2017). Cell type boundaries organize plant development. *elife* 6, e27421. 10.7554/eLife.27421.031 28895530 PMC5617630

[DEV198820C3] Chandler, J. W., Cole, M., Flier, A., Grewe, B. and Werr, W. (2007). The AP2 transcription factors DORNRÖSCHEN and DORNRÖSCHEN-LIKE redundantly control Arabidopsis embryo patterning via interaction with PHAVOLUTA. *Development* 134, 1653-1662. 10.1242/dev.001016 17376809

[DEV198820C4] Chandler, J. W., Jacobs, B., Cole, M., Comelli, P. and Werr, W. (2011). DORNRÖSCHEN-LIKE expression marks Arabidopsis floral organ founder cells and precedes auxin response maxima. *Plant Mol. Biol.* 76, 171-185. 10.1007/s11103-011-9779-8 21547450

[DEV198820C5] Cole, M., Chandler, J., Weijers, D., Jacobs, B., Comelli, P. and Werr, W. (2009). DORNROSCHEN is a direct target of the auxin response factor MONOPTEROS in the Arabidopsis embryo. *Development* 136, 1643-1651. 10.1242/dev.032177 19369397

[DEV198820C6] Cole, M., Jacobs, B., Soubigou-Taconnat, L., Balzergue, S., Renou, J. P., Chandler, J. W. and Werr, W. (2013). Live imaging of DORNRÖSCHEN and DORNRÖSCHEN-LIKE promoter activity reveals dynamic changes in cell identity at the microcallus surface of Arabidopsis embryonic suspensions. *Plant Cell Rep.* 32, 45-59. 10.1007/s00299-012-1339-4 23011125

[DEV198820C7] de Reuille, P. B., Bohn-Courseau, I., Ljung, K., Morin, H., Carraro, N., Godin, C. and Traas, J. (2006). Computer simulations reveal properties of the cell-cell signaling network at the shoot apex in Arabidopsis. *Proc. Natl. Acad. Sci. USA* 103, 1627-1632. 10.1073/pnas.0510130103 16432202 PMC1360567

[DEV198820C8] Eshed, Y., Izhaki, A., Baum, S. F., Floyd, S. K. and Bowman, J. L. (2004). Asymmetric leaf development and blade expansion in Arabidopsis are mediated by KANADI and YABBY activities. *Development* 131, 2997-3006. 10.1242/dev.01186 15169760

[DEV198820C9] Goldshmidt, A., Alvarez, J. P., Bowman, J. L. and Eshed, Y. (2008). Signals derived from YABBY gene activities in organ primordia regulate growth and partitioning of Arabidopsis shoot apical meristems. *Plant Cell* 20, 1217-1230. 10.1105/tpc.107.057877 18469164 PMC2438466

[DEV198820C10] Hamant, O., Das, P. and Burian, A. (2019). Time-lapse imaging of developing shoot meristems using a confocal laser scanning microscope. *Methods Mol. Biol.* 1992, 257-268. 10.1007/978-1-4939-9469-4_17 31148044

[DEV198820C11] Heisler, M. G., Ohno, C., Das, P., Sieber, P., Reddy, G. V., Long, J. A. and Meyerowitz, E. M. (2005). Patterns of auxin transport and gene expression during primordium development revealed by live imaging of the Arabidopsis inflorescence meristem. *Curr. Biol.* 15, 1899-1911. 10.1016/j.cub.2005.09.052 16271866

[DEV198820C12] Heisler, M. G., Hamant, O., Krupinski, P., Uyttewaal, M., Ohno, C., Jönsson, H., Traas, J. and Meyerowitz, E. M. (2010). Alignment between PIN1 polarity and microtubule orientation in the shoot apical meristem reveals a tight coupling between morphogenesis and auxin transport. *PLoS Biol.* 8, e1000516. 10.1371/journal.pbio.1000516 20976043 PMC2957402

[DEV198820C13] Hervieux, N., Dumond, M., Sapala, A., Routier-Kierzkowska, A.-L., Kierzkowski, D., Roeder, A. H. K., Smith, R. S., Boudaoud, A. and Hamant, O. (2016). A mechanical feedback restricts sepal growth and shape in *Arabidopsis*. *Curr. Biol.* 26, 1019-1028. 10.1016/j.cub.2016.03.004 27151660

[DEV198820C14] Husbands, A. Y., Chitwood, D. H., Plavskin, Y. and Timmermans, M. C. P. (2009). Signals and prepatterns: new insights into organ polarity in plants. *Genes Dev.* 23, 1986-1997. 10.1101/gad.1819909 19723761 PMC2751976

[DEV198820C15] Kerstetter, R. A., Bollman, K., Taylor, R. A., Bomblies, K. and Poethig, R. S. (2001). KANADI regulates organ polarity in Arabidopsis. *Nature* 411, 706-709. 10.1038/35079629 11395775

[DEV198820C16] Kierzkowski, D., Lenhard, M., Smith, R. and Kuhlemeier, C. (2013). Interaction between meristem tissue layers controls phyllotaxis. *Dev. Cell* 26, 616-628. 10.1016/j.devcel.2013.08.017 24091013

[DEV198820C17] Koch, A. J. and Meinhardt, H. (1994). Biological pattern formation: from basic mechanisms to complex structures. *Rev. Mod. Phys.* 66, 1481-1507. 10.1103/RevModPhys.66.1481

[DEV198820C18] Kuhlemeier, C. and Timmermans, M. C. P. (2016). The Sussex signal: insights into leaf dorsiventrality. *Development* 143, 3230-3237. 10.1242/dev.131888 27624828

[DEV198820C19] McConnell, J. R., Emery, J., Eshed, Y., Bao, N., Bowman, J. and Barton, M. K. (2001). Role of PHABULOSA and PHAVOLUTA in determining radial patterning in shoots. *Nature* 411, 709-713. 10.1038/35079635 11395776

[DEV198820C20] Nakata, M., Matsumoto, N., Tsugeki, R., Rikirsch, E., Laux, T. and Okada, K. (2012). Roles of the middle domain-specific WUSCHEL-RELATED HOMEOBOX genes in early development of leaves in Arabidopsis. *Plant Cell* 24, 519-535. 10.1105/tpc.111.092858 22374393 PMC3315230

[DEV198820C21] Qi, J., Wang, Y., Yu, T., Cunha, A., Wu, B., Vernoux, T., Meyerowitz, E. and Jiao, Y. (2014). Auxin depletion from leaf primordia contributes to organ patterning. *Proc. Natl. Acad. Sci. USA* 111, 18769-18774. 10.1073/pnas.1421878112 25512543 PMC4284573

[DEV198820C22] Refahi, Y., Zardilis, A., Michelin, G., Wightman, R., Leggio, B., Legrand, J., Faure, E., Vachez, L., Armezzani, A., Risson, A.-E. et al. (2021). A multiscale analysis of early flower development in Arabidopsis provides an integrated view of molecular regulation and growth control. *Dev. Cell* 56, 540-556.e8. 10.1016/j.devcel.2021.01.019 33621494 PMC8519405

[DEV198820C23] Reinhardt, D., Pesce, E.-R., Stieger, P., Mandel, T., Baltensperger, K., Bennett, M., Traas, J., Friml, J. and Kuhlemeier, C. (2003). Regulation of phyllotaxis by polar auxin transport. *Nature* 426, 255-260. 10.1038/nature02081 14628043

[DEV198820C24] Reinhardt, D., Frenz, M., Mandel, T. and Kuhlemeier, C. (2005). Microsurgical and laser ablation analysis of leaf positioning and dorsoventral patterning in tomato. *Development* 132, 15-26. 10.1242/dev.01544 15563522

[DEV198820C25] Sawa, S., Watanabe, K., Goto, K., Liu, Y. G., Shibata, D., Kanaya, E., Morita, E. H. and Okada, K. (1999). FILAMENTOUS FLOWER, a meristem and organ identity gene of Arabidopsis, encodes a protein with a zinc finger and HMG-related domains. *Genes Dev.* 13, 1079-1088. 10.1101/gad.13.9.1079 10323860 PMC316944

[DEV198820C26] Scanlon, M. J., Schneeberger, R. G. and Freeling, M. (1996). The maize mutant *narrow sheath* fails to establish leaf margin identity in a meristematic domain. *Development* 122, 1683-1691. 10.1242/dev.122.6.1683 8674408

[DEV198820C27] Segonzac, C., Nimchuk, Z. L., Beck, M., Tarr, P. T., Robatzek, S., Meyerowitz, E. M. and Zipfel, C. (2012). The shoot apical meristem regulatory peptide CLV3 does not activate innate immunity. *Plant Cell* 24, 3186-3192. 10.1105/tpc.111.091264 22923673 PMC3462624

[DEV198820C28] Shi, J., Dong, J., Xue, J., Wang, H., Yang, Z., Jiao, Y., Xu, L. and Huang, H. (2017). Model for the role of auxin polar transport in patterning of the leaf adaxial-abaxial axis. *Plant J.* 92, 469-480. 10.1111/tpj.13670 28849614

[DEV198820C29] Shi, B., Wang, H. and Jiao, Y. (2020). Live imaging of arabidopsis axillary meristems. *Methods Mol. Biol.* 2094, 59-65. 10.1007/978-1-0716-0183-9_7 31797291

[DEV198820C30] Siegfried, K. R., Eshed, Y., Baum, S. F., Otsuga, D., Drews, G. N. and Bowman, J. L. (1999). Members of the YABBY gene family specify abaxial cell fate in Arabidopsis. *Development* 126, 4117-4128. 10.1242/dev.126.18.4117 10457020

[DEV198820C31] Snow, R. and Snow, M. (1954a). Experiments on the cause of dorsiventrality in leaves. *Nature* 173, 644-644. 10.1038/173644a0

[DEV198820C32] Snow, R. and Snow, M. (1954b). Experiments on the cause of dorsiventrality in leaves. *Nature* 174, 352-353. 10.1038/174352a0

[DEV198820C33] Sussex, I. M. (1951). Experiments on the cause of dorsiventrality in leaves. *Nature* 167, 651-652. 10.1038/167651a0 14826895

[DEV198820C34] Tameshige, T., Fujita, H., Watanabe, K., Toyokura, K., Kondo, M., Tatematsu, K., Matsumoto, N., Tsugeki, R., Kawaguchi, M., Nishimura, M. et al. (2013). Pattern dynamics in adaxial-abaxial specific gene expression are modulated by a plastid retrograde signal during Arabidopsis thaliana leaf development. *PLoS Genet.* 9, e1003655. 10.1371/journal.pgen.1003655 23935517 PMC3723520

[DEV198820C35] Watanabe, K. and Okada, K. (2003). Two discrete cis elements control the Abaxial side-specific expression of the FILAMENTOUS FLOWER gene in Arabidopsis. *Plant Cell* 15, 2592-2602. 10.1105/tpc.015214 14555697 PMC280563

[DEV198820C36] Xu, T.-T., Ren, S.-C., Song, X.-F. and Liu, C.-M. (2015). CLE19 expressed in the embryo regulates both cotyledon establishment and endosperm development in Arabidopsis. *J. Exp. Bot.* 66, 5217-5227. 10.1093/jxb/erv293 26071532 PMC4526921

[DEV198820C37] Yamaguchi, T., Nukazuka, A. and Tsukaya, H. (2012). Leaf adaxial-abaxial polarity specification and lamina outgrowth: evolution and development. *Plant Cell Physiol.* 53, 1180-1194. 10.1093/pcp/pcs074 22619472

[DEV198820C38] Zhang, C., Wang, J., Wenkel, S., Chandler, J. W., Werr, W. and Jiao, Y. (2018). Spatiotemporal control of axillary meristem formation by interacting transcriptional regulators. *Development* 145, dev158352. 10.1242/dev.158352 30446629 PMC6307885

[DEV198820C39] Zhao, F., Chen, W., Sechet, J., Martin, M., Bovio, S., Lionnet, C., Long, Y., Battu, V., Mouille, G., Monéger, F. et al. (2019). Xyloglucans and microtubules synergistically maintain meristem geometry and phyllotaxis. *Plant Physiol.* 181, 1191-1206. 10.1104/pp.19.00608 31537749 PMC6836833

[DEV198820C40] Zhao, F., Du, F., Oliveri, H., Zhou, L., Ali, O., Chen, W., Feng, S., Wang, Q., Lü, S., Long, M. et al. (2020). Microtubule-mediated wall anisotropy contributes to leaf blade flattening. *Curr. Biol.* 30, 3972-3985.e6. 10.1016/j.cub.2020.07.076 32916107 PMC7575199

[DEV198820C41] Zhu, M., Chen, W., Mirabet, V., Hong, L., Bovio, S., Strauss, S., Schwarz, E. M., Tsugawa, S., Wang, Z., Smith, R. S. et al. (2020). Robust organ size requires robust timing of initiation orchestrated by focused auxin and cytokinin signalling. *Nat. Plants* 6, 686-698. 10.1038/s41477-020-0666-7 32451448 PMC7299778

