# Supplementary material for: Stable establishment of organ polarity occurs several plastochrons before primordium outgrowth in Arabidopsis
Source: Development. 2021 Jun 16;148(18):dev198820. doi: 10.1242/dev.198820 (PMC8255034; doi:10.1242/dev.198820)
Supplement: Supplementary information [file develop-148-198820-s1.pdf]

Supplementary information

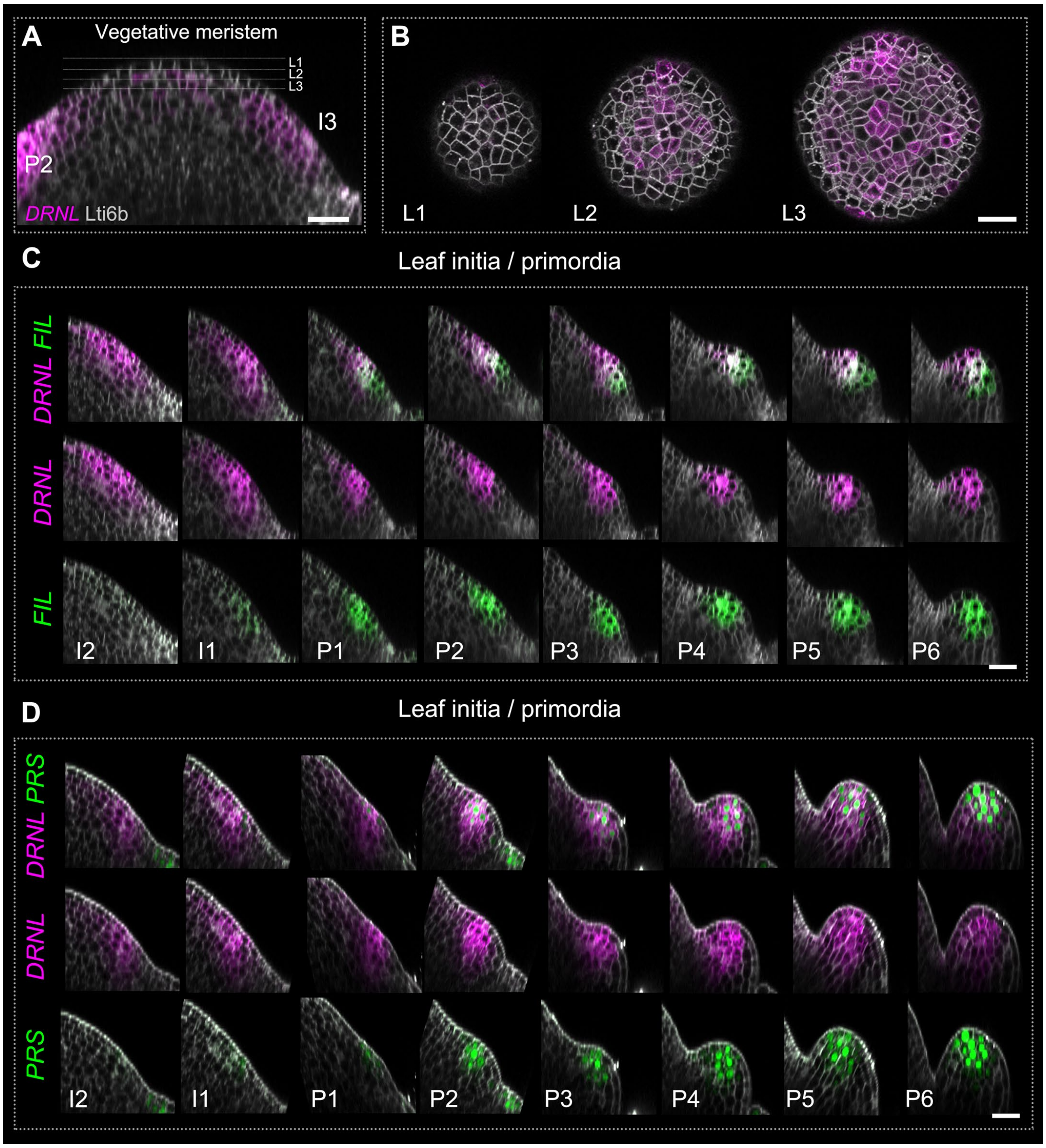

Figure S1. Gene expression pattern in leaf initia/primordia. Related to Figure 1.

(**A**) *pDRNL* activity in the longitudinal section of vegetative meristem.

(**B**) The top view of the *pDRNL* signals at different cell layers from the meristem dome, showing very patchy expression of *DRNL* in L2 and L3 layers. The cross-sections were along the cell planes indicated in (A).

(**C**) Longitudinal sections of leaf initia/primordia from I_2 to P_6. This developmental

Development • Supplementary information

series is taken from the same meristem at the same time point. *pDRNL* is initially activated in a large area at the flank of the meristem, then is restricted to the adaxial and middle domain of the leaf primordia from P_1 onwards until P_6. On the contrary, *pFIL* expression initially mostly overlaps with *pDRNL* and then gradually shifts abaxially from P_1 onwards. At P_6, *pFIL* signals are located at the abaxial and middle domains.

(**D**) Longitudinal sections of leaf initia/primordia from I_2 to P_6 showing the colocalization of *pDRNL* and *pPRS*. *pPRS* is initially activated within the *pDRNL* domain at I_1. Later on, *pPRS* signals shift from the adaxial to the middle domain (from P_1 to P_4) and remain restricted to the ad-abaxial boundary. Scale bars: 20µm.

Development • Supplementary information

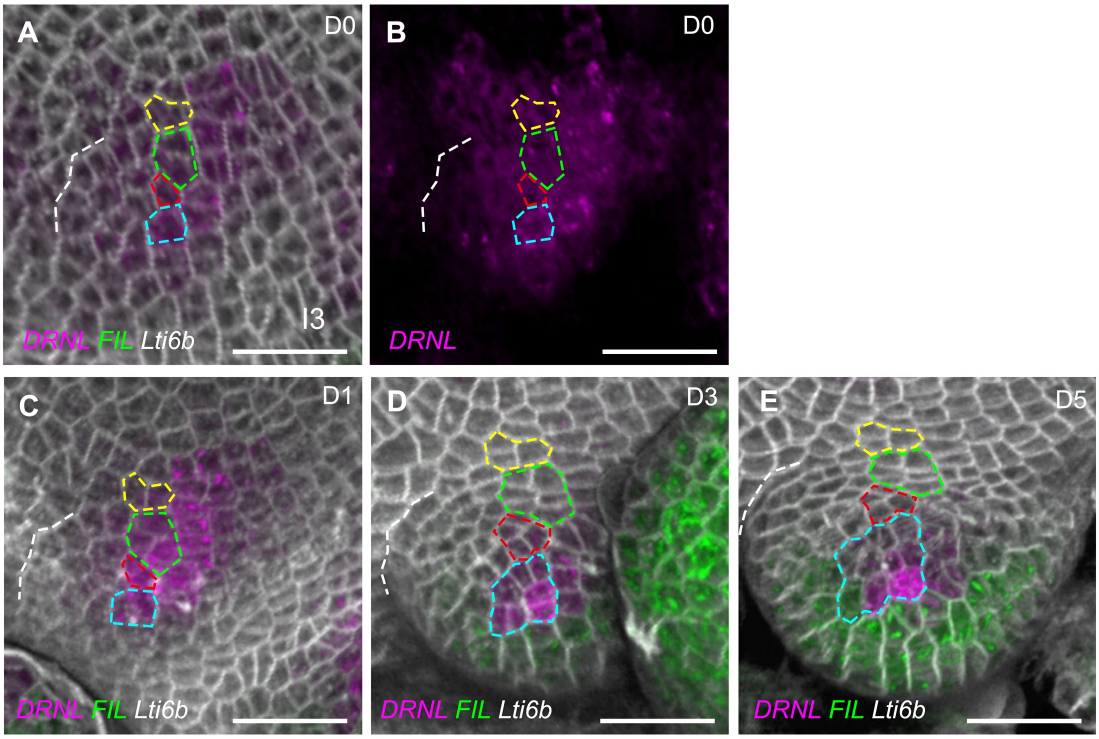

Figure S2. Detailed cell lineage tracking at I_3 position in vegetative meristem. Related to Figure 1.

(**A**) Top view of 3D reconstruction of I_3 leaf initium expressing *pDRNL*, *pFIL* and membrane markers.

(**B**) *DRNL* channel of image in (A).

(**C-E**) The growth of the I_3 initium tracked for five days. Sister cells are surrounded by colored dotted lines. The dashed white line represents the lateral boundary of the leaf initium/primordium. Scale bars: 20µm.

Development • Supplementary information

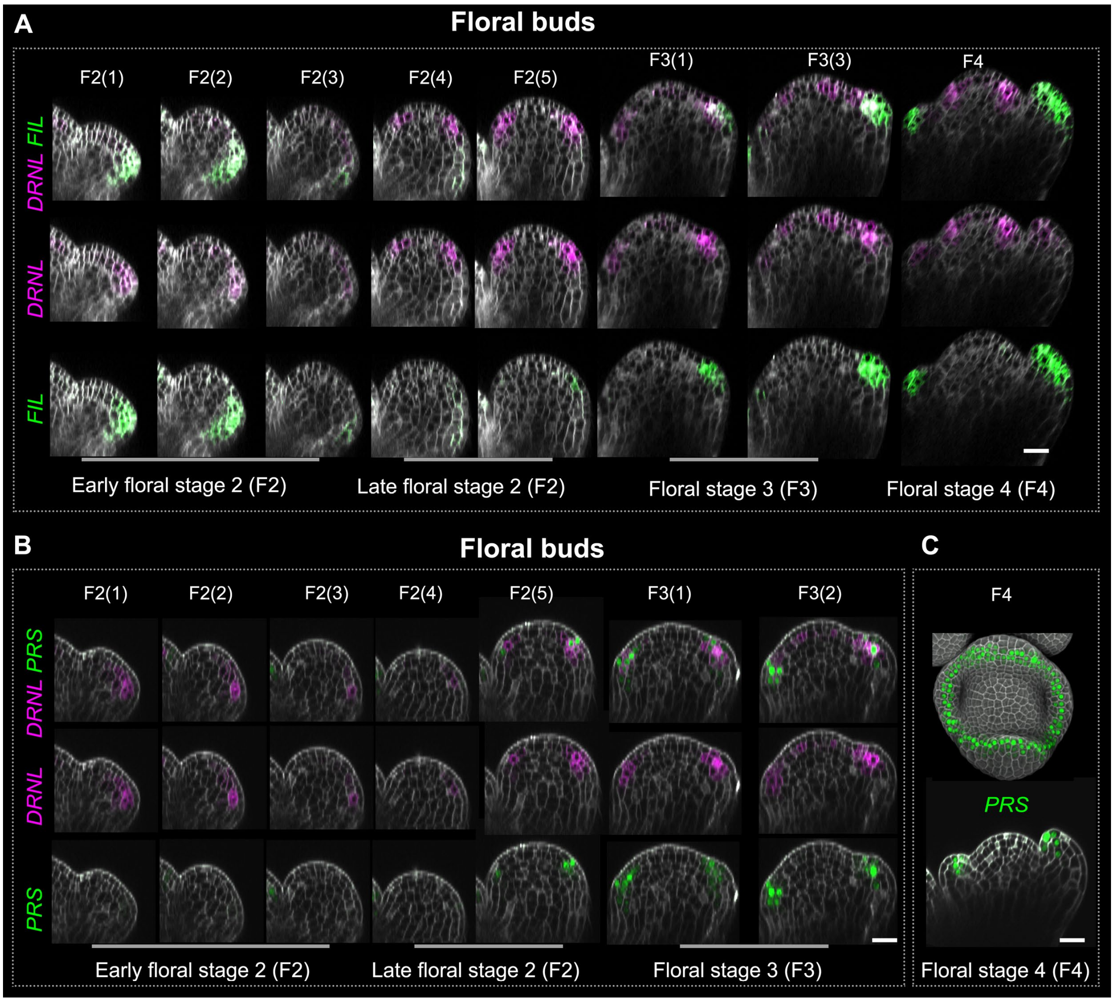

Figure S3. Gene expression pattern in floral buds. Related to Figure 2.

(**A**) Longitudinal sections of floral buds from stage 2 to stage 4 with detailed subdivisions (e.g. F2(1), F2(2) etc.). Developmental series taken from the same inflorescence. During early stage 2, the *pDRNL* and *pFIL* activation domains are largely overlapping in the cryptic bract. *pDRNL* and *pFIL* are gradually repressed in the cryptic bract, to be fully switched off at the beginning of late stage 2. At the same time, *pDRNL* is activated in sepal initia. As in the leaf, *pDRNL* signals are gradually restricted to the adaxial domain during floral stage 3, and to the sepal tip at floral stage 4. *pFIL* activity initially mostly overlaps with the *pDRNL* domain and then becomes more abaxial at floral stage 3 During floral stage 3, *pFIL* expression gradually shifts abaxially, and becomes located at the abaxial and middle domains at floral stage 4.

(**B**) Longitudinal sections of floral buds from stage 2 to stage 3. Note that there is no detectable *pPRS* signal in the cryptic bract during early stage 2. *pPRS* is activated during

Development • Supplementary information

late floral stage 2 in the future sepal, then becomes gradually restricted to the adaxial domains during floral stage 3.

(**C**) *pPRS* signals in stage 4 floral bud in 3D (upper panel) and section (lower panel). The *pPRS* signal finally shifts to the middle domain of the sepal at floral stage 4 (lower panel). Note that *pPRS* is also active in lateral sepal boundaries. Scale bars: 20µm.

Development • Supplementary information

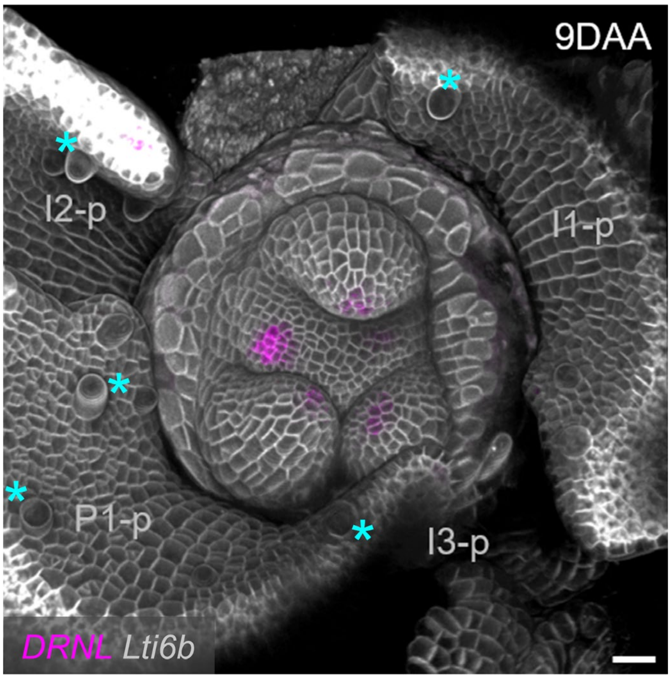

Figure S4. Architecture of vegetative meristem and leaf development at nine days after circular ablations. Related to Figure 3.

Top view of the vegetative meristem shown in Fig. 3 at nine days after ablation (9DAA). The ablation has caused a ring-like outgrowth. Note that leaves next to the ablation at the I_1 to P_1 positions (I_1-p to P_1-p) developed a clear polarity with trichomes (blue stars) on the adaxial side and have grown substantially (compare with Fig. 3B). New primordia (expressing *pDRNL:erCERULEAN)* are forming inside the healed wound. There was no leaf formed at I_3 position (I_3-p). Scale bar: 20μm.

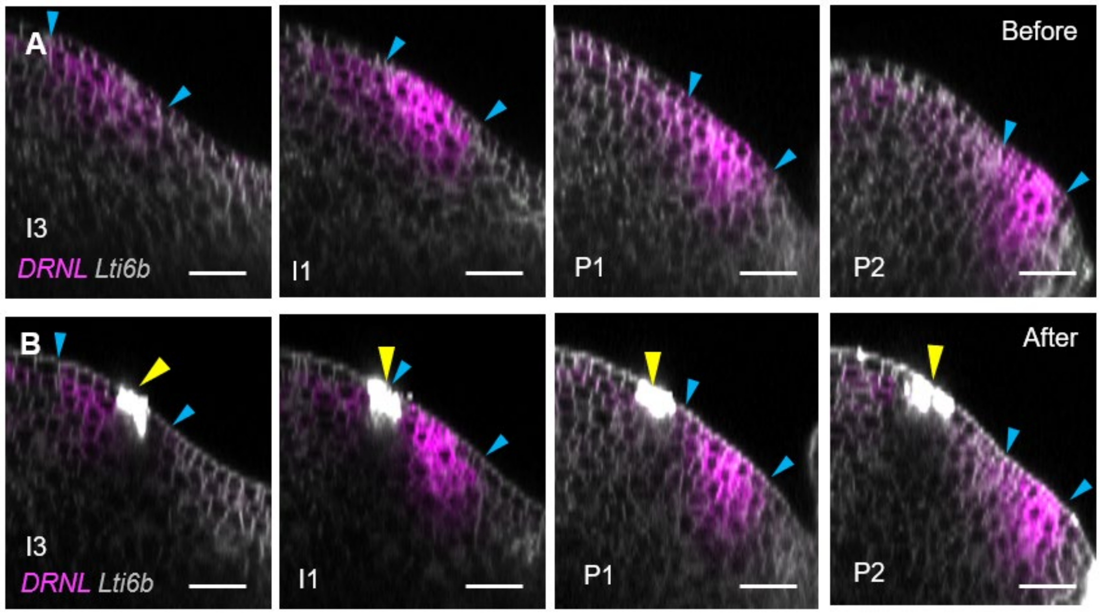

Figure S5. Wounding positions after circular ablations in the vegetative meristem shown in Figure 3.

(**A-B**) Longitudinal sections at different positions in the meristem (shown in Figure 3A) before (A) and after ablation (B). The wounds are indicated by yellow arrowheads. The expression domains of *pDRNL* are indicated by blue arrowheads. Scale bars: 20µm.

Development • Supplementary information

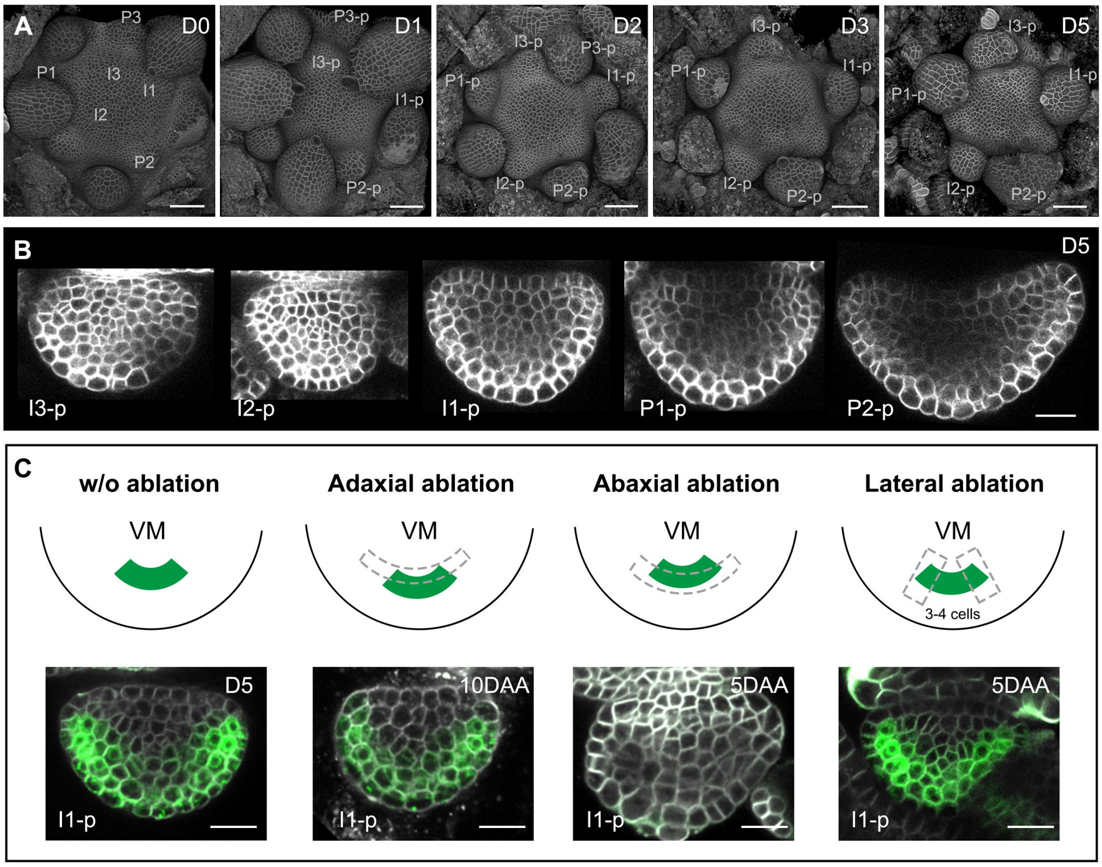

Figure S6. Leaf shape and polarity in intact and wounded vegetative meristems.

(**A**) A representative intact vegetative meristem cultured *in vitro* for 5 days.

(**B**) Cross-sections of leaf primordia at different positions on day 5.

(**C**) leaf polarity in control and after different types of ablations. The abaxial identity is marked by *pFIL* signals (green). Scale bars: 50µm in (A); 20µm in (B and C).

Development • Supplementary information

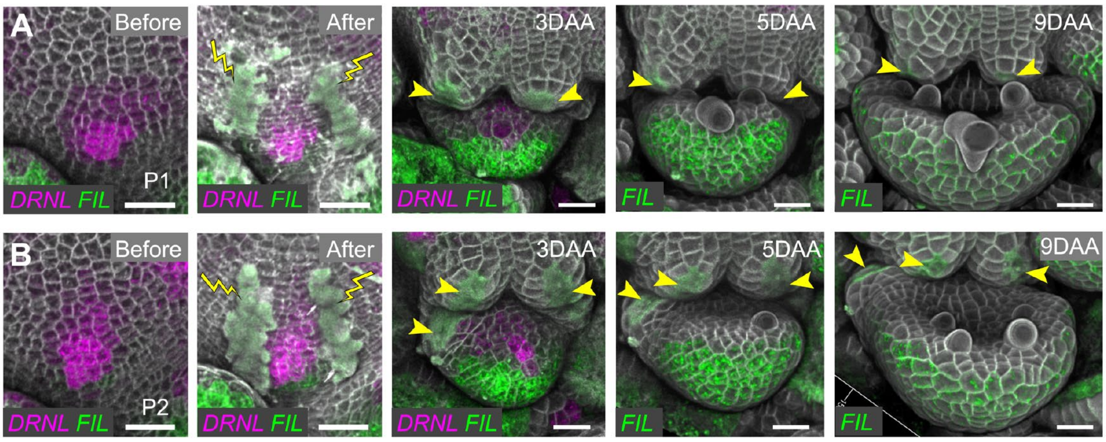

Figure S7. Lateral ablations at P1 and P2. Related to Figure 4.

(A-B) Representative lateral ablations at P1 (A) and P2 (B), 4-5 cells wide. The wounds are marked by yellow lightning signs and arrowheads. After the ablations, the growth was followed for 9 days Scale bars: 20µm.

Development • Supplementary information

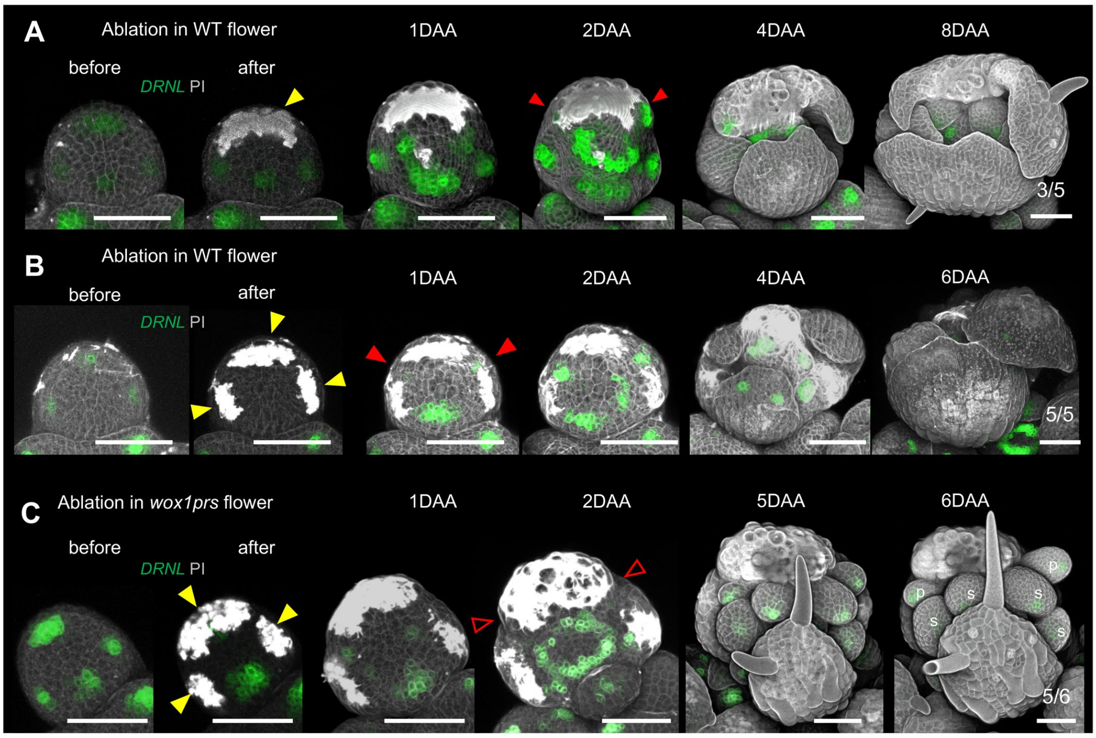

Figure S8. Regeneration of sepal primordia requires *WOX1/PRS* activity.

(**A**) When founder cells (indicated by *pDRNL:erGFP*) of the outer sepal were killed, new founder cells were regenerated from two lateral boundaries of the eliminated primordia (red arrowheads). (**B**) The elimination of founder cells of the outer and two lateral sepals in wild type flowers. The regeneration occurs de novo at lateral boundaries (red arrowheads) indicating that regeneration is not induced by the signal from neighboring sepal primordia. The same results were obtained from 5 different floral buds.

(**C**) The elimination of founder cells of the outer and two lateral sepals in a *wox1 prs* flower. There is no regeneration of new sepal primordia at lateral boundaries (hollow red arrowheads). p, petal; s, stamen. The same results were obtained from 5 meristems. The wounds are marked by yellow arrowheads in (A-C). Scale bars: 50µm.

Development • Supplementary information
