# Supplementary material for: Stable establishment of organ polarity occurs several plastochrons before primordium outgrowth in Arabidopsis
Source: Development. 2021 Jun 16;148(18):dev198820. doi: 10.1242/dev.198820 (PMC8255034; doi:10.1242/dev.198820)
Supplement: Reviewer comments [file develop-148-198820_review_history.pdf]

Stable establishment of organ polarity occurs several plastochrons before primordium outgrowth in *Arabidopsis*

Feng Zhao and Jan Traas
DOI: 10.1242/dev.198820

Editor: Ykä Helariutta

Review timeline

Original submission:	20 November 2020
Editorial decision:	11 January 2021
First revision received:	19 March 2021
Editorial decision:	16 April 2021
Second revision received:	28 April 2021
Accepted:	6 May 2021

Original submission

First decision letter

MS ID#: DEVELOP/2020/198820

MS TITLE: Stable establishment of organ polarity several plastochrones before primordium outgrowth in Arabidopsis

AUTHORS: Feng Zhao and Jan Traas

I have now received all the referees reports on the above manuscript, and have reached a decision. The referees' comments are appended below, or you can access them online: please go to BenchPress and click on the 'Manuscripts with Decisions' queue in the Author Area.

The overall evaluation is positive and we would like to publish a revised manuscript in Development, provided that the referees' comments can be satisfactorily addressed. Please attend to all of the reviewers' comments in your revised manuscript and detail them in your point-by-point response. If you do not agree with any of their criticisms or suggestions explain clearly why this is so.

We are aware that you may currently be unable to access the lab to undertake experimental revisions. If it would be helpful, we encourage you to contact us to discuss your revision in greater detail. Please send us a point-by-point response indicating where you are able to address concerns raised (either experimentally or by changes to the text) and where you will not be able to do so within the normal timeframe of a revision. We will then provide further guidance. Please also note that we are happy to extend revision timeframes as necessary.

Reviewer 1

Advance summary and potential significance to field

Plant biologists' speculation that a signal from the meristem establishes the polarity of the organ is based on classic experiments by Sussex. However, recent work suggests polarity is established as a prepattern when the organ forms, and the prepattern is disrupted in response to ablation rather than from loss of signal from the meristem (Caggiano, 2017). In this manuscript, Zhao and Traas

© 2021. Published by The Company of Biologists under the terms of the Creative Commons Attribution License (https://creativecommons.org/licenses/by/4.0/).

show that their ablations fail to cause the formation of radialized organs (leaves or sepals lacking polariy) in Arabidopsis. They show beautiful live imaging which tracks the expression of organ founder (DRNL) and polarity markers (FIL and PRS) in wild type vegetative and floral meristems. Interestingly, their imaging shows that the domains of DRNL and FIL expression move across cells as the organ grows out, such that the cells initially expressing the markers are not the same ones that express the markers in the primordium. This result parallels the observations in Galvan-Ampudia et al. (eLife 2020;9:e55832)that the auxin maxima establishing the primordium moved across cells.

To test the effects of ablation on polarity, the authors make circular ablations around the meristem, isolating it from incipient primordia, and live image development over time. They show that the existing incipient primordia form normal polarity despite the ablation. They also use lateral ablations previously shown to cause radialized leaves in tomato, but these also did not cause radialized leaves to form in Arabidopsis. Then they ablate the adaxial domains, yet still the leaves manage to re-establish their polarity. Next, they ablate the abaxial domain and growth halts. They do more ablation experiments where they show they are able to reduce or stop growth, but still do not generate radialized leaves. Finally, they ablate sepals entirely and show that new sepal primordia with proper polarity form from the margins. However, they show the regeneration of sepals from margins fails in prs wox1 mutants. In their introduction they present a concise yet thorough historical context for this manuscript. We think the detailed live imaging will be of interest to the plant development community. They effectively used their characterization of marker expression patterns to be able to control the timing of their ablations more precisely than based on morphology alone.

Comments for the author

The challenge with this manuscript is that it essentially presents negative data, i.e. the ablations do not cause a change in polarity, which raises the question of whether their ablations worked. How many cell layers deep were the ablations? While it is true that ablation of the L1 is sufficient to cause radialized leaves in tomatoes it is possible Arabidopsis might require a deeper ablation. Are circular ablations too strong? The later images show trichomes developing on the flanks of the meristem and severe disruption. In the Caggiano paper, an ablation in the Arabidopsis meristem can cause the abaxial polarity factor KANADI1 to completely encircle the primordium, so it is a bit surprising that the ablations here would not cause any change in polarity. Zhao and Traas do point out that Caggiano did not track the development of that organ to determine its eventual polarity. Could it be possible that ablations do cause this change in KANADI, but that polarity is re-established to have proper FIL expression? How do you determine whether polarity maintained or reorganized? It would be useful to establish wether the ablations shown in the paper alter KANADI expression or not. It would also be useful show the alterations of the FIL and PRS expression patterns in a radialized Arabidopsis organ (which do form in mutants) as a control for comparison.

The writing of the paper is a bit dense. The paper has so much detailed description that the main points sometimes get lost. It would help to add some more diagrams to orient readers, particularly those not familiar with plant meristems.
It seemed like the wox1 prs double mutant in Figure 9 was a bit extraneous and could be removed from the paper or put in the supplement.

<u>Reviewer 2</u>

Advance summary and potential significance to field

Zhao and Traas revisits a classical set of surgical experiments into the origin of leaf dorsiventrality that were originally performed by Sussex on potato and subsequently by Reinhardt et al. on tomato meristems. Here, incisions that separate the new to emerge primordium from the remainder of the meristem resulted in radial, abaxialized leaf primordia. The basis for this phenotype remains a topic of debate. In addition, it remained open whether this phenotype translates into species outside the Solanaceae. The manuscript describes outcomes from surgical ablations on the vegetative and floral apex of Arabidopsis that express early organ and polarity markers to precisely set the site of ablation, and to follow the outcome on dorsiventrality.

© 2021. Published by The Company of Biologists under the terms of the Creative Commons Attribution License (https://creativecommons.org/licenses/by/4.0/).

The authors report that none of their surgical experiments led to formation of radialized organs, even when incisions were made on incipient primordia several plastochrons prior to emergence. They therefore conclude that organ polarity is established in early incipient primordia and is stable despite major perturbations at the meristem. The manuscript can make a nice addition to Development. However, several key points need to be resolved to fully substantiate the conclusions drawn.

Comments for the author

The authors trace cell lineages starting from I1. DRN then mainly marks the meristem-organ boundary, but subsequently DRN localizes to adaxial side and middle domain. Given that the observed patterns of expression are not stable over primordium development, what is the fate of cells marked by DRN at I3? Does DRN at this early stage mark the primordium boundary only or also organ founder cells? If the latter, all founder cells or only those cells of the future adaxial side? This is important to understand the position of the incisions relative to the developing primordium.

Likewise for the floral meristems shown in Figure 2. Cells marked with an arrowhead remain at the meristem. Does DRN mark sepal founder cells in stage 2 and stage 3 flowers, or is the DRN expression domain positioned at the meristem-organ boundary. It would be more informative to also show the fate of cells at the abaxial boundary of the DRN expression domain.

In the earlier surgical experiments, the position of the incision was critical to observing polarity defects. Indeed, in tomato, only a subset of primordia show a loss of organ polarity (Reinhardt et al, 2005). Therefore, a more in depth analysis/description of the exact position of the ablations relative to the DRN expressing cells and even more so relative to the future primordium (see the point above) is needed for each I3-P2. Information is provided for a I2 primordium in Fig. 3. Where were the incisions positioned relative to I3 and I1, and likewise relative to P1 and P2? Optical sections and a diagram reminiscent of Fig. 5A for each primordial stage would be needed to truly compare the observations made here to those from the earlier work. In that regard, was the position of the ablation identical across the 12 meristems analyzed?

The same point applies for the lateral ablations at leaf primordia and the incisions at sepal primordia. More information is needed on the position of these incisions in relation to what would be the fate of the cells.

Likewise, more careful descriptions are needed to interpret the outcomes of the manipulations. For example, in figure 3C, a comparison to primordia on intact meristems is absolutely critical. In addition, expression information from FIL and PRS is needed to demonstrate more convincingly that ad-abaxial polarity is unperturbed. Particularly so in the youngest primordia.

However, the most critical point is that for quite a percentage of primordia the effects of the manipulations are not described. In this first surgical experiment, what happens in the remaining 8/12 I3 primordia, 4/8 I2 primordia, and 2/12 I1 primordia? In this and the subsequent experiments quite some primordia are reported to arrest growth. The basis for this is not addressed. This point is particularly relevant as growth seems to arrest with a few days delay from the manipulations (e.g. Figs. 4B, 6C, 7A, 7B) and expression of FIL and DRN is lost. What follows what? Thinking about the surgical experiments shown in Fig. 6 and the statement on page 7, that some leaf primordia with limited growth show extended FIL expression, it needs to be addressed whether loss of ad-abaxial polarity underlies the growth arrest, or whether the reverse is the case. Again, at least for the surgical experiments in tomato, only a subset of surgically-isolated primordia showed a polarity defect.

Related to this, ablation of the adaxial side of primordia reorients growth leading to establishment of a new meristem-organ boundary below the wound (page 6). Can it be excluded that this new boundary be responsible for ad-abaxial polarity in the subtending primordium? Figure 4D also shows additional growth between the incision and the primordium below. How might this contribute to the lack of phenotypes observed?

© 2021. Published by The Company of Biologists under the terms of the Creative Commons Attribution License (https://creativecommons.org/licenses/by/4.0/).

In short, a more comprehensive careful analysis/description of the manipulations and the phenotypes that follow is needed to allow a full assessment of effects on ad-abaxial polarity.

Additional points:
For figure 7C, it is stated that polarity is not affected up to 6 DAA (although it is subsequently). The first is not obvious from the data shown. Transverse FIL sections compared to normal primordia would be informative. As mentioned above, such data and comparisons to normal primordia are also need in other figures.

The labeling in Fig 5 is not entirely legible.

Please include also a top view of the incisions reported in Figure 6. Here it should be noted that Sussex reports that smaller incisions at primordia that fail to fully isolate it from the rest of the meristem do not perturb dorsiventrality.

Reviewer 3

Advance summary and potential significance to field

The authors report a valuable dataset on the important question of mechanisms underlying establishment of tissue polarity in shoot lateral organs thus revisiting ideas first reported by Sussex in the 1950s and also discussed and investigated by the SnowÂ’s in the same decade and other researchers in the modern era.

The authors use Arabidopsis thaliana and broadly speaking the logic of the work is to use ablation of selected areas in initiating primordia (e.g. adaxial or abaxial) to determine effects on tissue polarity. These experiments can help inform on the timing of polarity establishment and also can help understand whether signals travel between the shoot apical meristem and lateral organ primordia and vice versa. The authors use tissue polarity marker expression to aid selection of cells that are to be ablated.

Overall the authors propose that unlike what has been seen solanaceous plants in their system tissue polarity is fixed very early and cannot be perturbed with ablations.

They discuss their observations in light of other recent efforts to conduct similar experiments and also consider the possible effects of wounding responses that can confound interpretation of ablations. It is also of note that this work to some extent overcome the drawbacks of similar experiments performed in very young seedlings (Caggiano et al 2017), where a long- term monitoring of the ablated growth primordium was not possible. Also, the findings that regeneration of sepals post ablation requires WOX activity are novel and interesting.

Comments for the author

It might be that different choices of markers genes used to guide ablation or monitor consequences thereof would have yielded different results. However, these types of experiments are non-trivial and only so much can be done for one study so I think the appropriate path is to to rethink the data under this perspective and when needed recast interpretations given the points below.

1 At inception FIL has been shown to express in both the abaxial (away from the shoot axis) and adaxial regions of the primordium at inception (Caggiano et al 2017, Tamashige et al 2013). Also, FIL doesn’t show expression adaxial to P1, coinciding with the zone of PIN1 polarity reversal at P1 stage (also seen in this study and in agreement with prior work). FIL becomes more abaxial when the leaf primordium has grown out of the meristem so at quite a late stage. Work from Y. Eshed J Bowman has indicated that Yabby’s are likely predominantly involved in growth rather than tissue polarity (Eshed et al Development 2004 131:2997-3006; doi: 10.1242/dev.01186) Also please see Goldschidt et al Plant Cell 2008 May;20(5):1217-30. doi: 10.1105/tpc.107.057877. Epub 2008 May 9 on YABBY related signalling that merits more discussion here. The conclusion from all this is that FIL may not be a typical abaxial marker like KANADI and that more specific conclusions are needed that incorporate more nuanced views on how FIL functions.

© 2021. Published by The Company of Biologists under the terms of the Creative Commons Attribution License (https://creativecommons.org/licenses/by/4.0/).

2. Similarly regarding the choice of adaxial cell-type marker DRN, Figure 1D (D3) shows DRN expression in the boundary between the meristem and the initiating leaf where the dorsal marker REV would be absent and KANADI expression would re-establish, creating a new boundary for the next primordium to initiate. Thi point needs discussion. Related In the same figure, stage at day 5 shows pDRN expression mainly in the pro-
vasculature and subepidermal area as well as an absence from the dorsal cells. This doesn't quite match with data shown in Figure S1. What do the authors think on this? If this cannot be resolved with more data or more careful analysis of staging in existing data then maybe more replicates/more detailed substance could help address this variation in the future and this point can be clarified in discussion.

3. This data set as well as previous ones using ablations lack precise tracing of dorsal ventral cell lineages using dorso-ventral markers to really explain the outcome for organ polarity. Given their set-up (bigger meristems) and ability to monitor ablated primordia over time this would be possible here. This path could be highlighted in the discussion as a future step for the field.
The following related points pertain to specific interpretations provided.

4. Line 122- No apparent polarity is distinguishable before P1 when the primordia start to bulge out.
According to data based on live imaging, polarity is clear since the onset of the leaf primordium inception:
There may be some cell-level fluctuations (Yu et al 2017) but overall, the dorsoventral boundary appears to remain robust, unless perturbed by changes in auxin levels (Caggiano et al 2017). Please discuss accordingly.

5. Line172 ablations were performed at I3, when pDRNL was only just becoming upregulated and pFIL was not expressed at all, leaf polarity was not perturbed (Fig 3E). Given the REV is established earlier than DRN, REV KAN and PIN1 markers would have been important when trying to estimate the relative stages of primordia and the establishment of the ad/ab boundaries. The current images do not show if the wound is already passing through the KAN expression domain surrounding the primordium (for example this would be the case in P1 and P2 where DV boundaries should have stabilized by this stage). Please discuss accordingly.

6. The tangential ablations to disrupt adaxial and abaxial sides of the primordia are very good. However, since FIL shows expression in adaxial cells at very early stages, in this experiment, it may be that not all adaxial cells were ablated. Wound-induced KAN expression adaxial to some of the left over adaxial cells could have led to re-estabishment of the ad-ab boundary. The lineages of the remaining adaxial cells may have been sufficient to grow further and still maintain the ad-ab polarity of the ablated organ. That may explain the absence of polarity defects in the regenerating primordium. Also, it seems that the wound is not deep enough which may have contributed to the effects seen. Please discuss.

7. Similar considerations apply to the ablations performed in floral meristem as shown in Figure 8. One curious thing in Fig 8C, is that it seems there is ectopic PRS expression upon wounding. PRS expression is promoted by auxin and regions surrounding wounds have been shown to be associated with low auxin levels.
What then explains ectopic PRS expression there? Is that also seen in the vegetative meristems?

Minor point

Please define plastochron the first time it is used: Readers not familiar with hoot development or plant biology will probably not know the term plastrochrones is I think French I suggest using the English "plastrochrons"

© 2021. Published by The Company of Biologists under the terms of the Creative Commons Attribution License (https://creativecommons.org/licenses/by/4.0/).

First revision

Author response to reviewers' comments

Reviewer 1 Comments for the author

1. *The challenge with this manuscript is that it essentially presents negative data, i.e. the ablations do not cause a change in polarity, which raises the question of whether their ablations worked. How many cell layers deep were the ablations? While it is true that ablation of the L1 is sufficient to cause radialized leaves in tomatoes, it is possible Arabidopsis might require a deeper ablation.*

We agree with the reviewer that the efficiency of laser ablation is essential for the experiment. We made the wounds as deep as possible by setting the laser at the highest intensity with 5 repetitions for each point (see methods). Under such conditions, our ablations are usually around 2-3 cells deep. This is shown in Fig.3, Fig.6 and Fig.8. We now have quantified the ablations in vegetative meristems and found that in 60 ablations 38 were 2-3 cells deep, 19 3-4 cells deep, and 3 1-2 cells deep. This is now mentioned in the methods section (p13 line 444-446). Considering that the majority of cells in the lateral organs originate from L2 at the meristems, we think our ablations are sufficiently efficient to isolate the organ initial cells from the meristem. Please note that the ablations do cause local changes in gene expression (loss of *FIL* for example) and local outgrowths, clearly illustrating that there is an effect. In addition, we have previously observed, using the same equipment, that this type of ablation causes local changes in cytoskeleton organisation (Zhao et al., 2019).

2. *Are circular ablations too strong? The later images show trichomes developing on the flanks of the meristem and severe disruption.*

The reviewer refers to the local outgrowths and formation of trichomes. Please note that the ectopic outgrowths, also present after lateral ablations, do not perturb polarity of neighbouring outgrowing organs (Fig 3 and 4). As for the trichomes, we realise that the figure we showed might have been misleading: the trichomes are not on the flanks of meristem but rather on the adaxial side of P1 leaf primordia (Fig.3B), which is also the case during normal development. To clarify this, we have added a later stage of the meristem and P1 leaf as a supplementary figure (Fig. S4).

Please note that our circular ablation also did not block the outgrowth of inner initia (Fig.3B and see also new Fig.S4). Therefore, we do not think the circular ablations disrupt patterning too severely.

3. *In the Caggiano paper, an ablation in the Arabidopsis meristem can cause the abaxial polarity factor KANADI1 to completely encircle the primordium, so it is a bit surprising that the ablations here would not cause any change in polarity. Zhao and Traas do point out that Caggiano did not track the development of that organ to determine its eventual polarity. Could it be possible that ablations do cause this change in KANADI, but that polarity is re- established to have proper FIL expression? How do you determine whether polarity maintained or reorganized? It would be useful to establish whether the ablations shown in the paper alter KANADI expression or not.*

We initially planned to use the *pKAN1:KAN1-2xGFP* and *pREV:REV-VENUS* lines for our ablations. However, in our hands, the signals of these two lines were too weak to be used as reference for setting the track of the ablations. To solve this problem, we chose the stronger *pFIL* marker. Ablation of the adaxial part of the *pFIL* domain led to an inactivation or at least a significant reduction of the *FIL* signal close to the wound. This was followed by the formation of a new organ boundary and upregulation of *pFIL* at the abaxial side of the outgrowing primordium. This is in contrast to unablated controls where *pFIL* expression remained active once it is activated. So, as Caggiano et al, we find that ablation perturbs polarity gene expression, but where they find the local activation of the abaxial marker *KAN1*,

© 2021. Published by The Company of Biologists under the terms of the Creative Commons Attribution License (https://creativecommons.org/licenses/by/4.0/).

we find the inactivation of the abaxial marker *FIL* (Fig. 6A'-A''). In addition, our result also shows that these ablations do not permanently wipe out local polarity, in contrast to what might happen in tomato or potato. This would overall suggest that local patterning is only transiently perturbed and restored afterwards. Finally, our data also suggest that cells expressing *FIL* are absolutely required for the formation of a polarized organ as no or very little growth occurs when the entire domain is wiped out (Fig. 6B-B''). We now discuss this point more explicitly (page 7- 8, and page 10-11).

5. It would also be useful to show the alterations of the FIL and PRS expression patterns in a radialized Arabidopsis organ (which do form in mutants) as a control for comparison.

We did not cross the markers into radialized mutants. However, *in situ* hybridization shows that both *FIL* and *PRS* expressions are altered in radialized organs (Nakata et al., 2012). This particular *pFIL:GFP* marker shows an expanded expression in a partially abaxialized leaf mutant (Timershige et al., 2013). We have added a remark on this issue (p4 line 129-133). We have used *PRS* here mainly to refine *DRNL* and *FIL* expression, and only in one ablation experiment (lateral ablation in floral meristems). In addition, we do not base our conclusions on the sole expression of these markers, but combine them with other proofs for polarity (flat leaf shape and trichomes).

6. The writing of the paper is a bit dense. The paper has so much detailed description that the main points sometimes get lost. It would help to add some more diagrams to orient readers, particularly those not familiar with plant meristems.

Thanks for the suggestions. We now add extra diagrams in Fig.6, Fig.7 and Fig.8 to ease the reading of our readers. There is also a new figure 9 to summarize all the ablation results.

7. It seemed like the wox1 prs double mutant in Figure 9 was a bit extraneous and could be removed from the paper or put in the supplement.

We were hesitating to keep this part in the story as it is indeed somewhat outside the main scope of the paper. Finally, we decided to keep it, not only because this result is intriguing (as also pointed out by another reviewer), but also further demonstrated the robustness of dorsiventrality at the Arabidopsis meristems. We therefore would propose to keep it, but moved it to the supplementary as suggested (new Fig. S7).

Reviewer 2 Comments for the Author:

1. The authors trace cell lineages starting from I1. DRN then mainly marks the meristem- organ boundary, but subsequently DRN localizes to adaxial side and middle domain. Given that the observed patterns of expression are not stable over primordium development, what is the fate of cells marked by DRN at I3? Does DRN at this early stage mark the primordium boundary only or also organ founder cells? If the latter, all founder cells or only those cells of the future adaxial side? This is important to understand the position of the incisions relative to the developing primordium.

This is an important point. *DRNL* was initially reported as a founder cell marker (Chandler et al., 2011). Functionally it is involved in organ initiation. A recent paper (Capua and Eshed 2017) showed that loss-function of the *DRNL* ortholog in tomato blocked the initiation of leaves. In agreement with this, we found that when the wounds went through the abaxial part of the *DRNL* expression domain, the initiation of leaves was halted (e.g. at I3, shown in Fig.3A-B).

However, *DRNL* expression itself is not limited to the organ founder cells. At the I1 position (Fig.1C-D), part of the *DRNL* expressing cells had offspring in the adaxial domain and the rest were left in the organ boundary. This is also the case for the I3 position. We now have added top views with lineage showing this (new Fig.S2), and discuss this in the text (p4 line145-147 and p6 line 185-189).

© 2021. Published by The Company of Biologists under the terms of the Creative Commons Attribution License (https://creativecommons.org/licenses/by/4.0/).

2. *Likewise for the floral meristems shown in Figure 2. Cells marked with an arrowhead remain at the meristem. Does DRN mark sepal founder cells in stage 2 and stage 3 flowers, or is the DRN expression domain positioned at the meristem-organ boundary. It would be more informative to also show the fate of cells at the abaxial boundary of the DRN expression domain.*

We also found when all *DRNL*-expressed cells were killed, no sepal could form along the ad-abaxial axis (e.g. renumbered Fig.S7A). This means at least part of the *DRNL* expressing cells is involved in sepal initiation.

The relative expression of marker genes during sepal initiation is similar to that in leaves. We have indicated cell lineage in Fig 2C-E and H to show that *DRNL* initially covers the adaxial boundary and even part of the remaining meristematic zone (future whorl 3) (p5 line 170- 173).

3. *In the earlier surgical experiments, the position of the incision was critical to observing polarity defects. Indeed, in tomato, only a subset of primordia show a loss of organ polarity (Reinhardt et al, 2005). Therefore, a more in depth analysis/description of the exact position of the ablations relative to the DRN expressing cells and even more so relative to the future primordium (see the point above) is needed for each I3-P2. Information is provided for a I2 primordium in Fig. 3. Where were the incisions positioned relative to I3 and I1, and likewise relative to P1 and P2? Optical sections and a diagram reminiscent of Fig. 5A for each primordial stage would be needed to truly compare the observations made here to those from the earlier work. In that regard, was the position of the ablation identical across the 12 meristems analyzed?*

As we mentioned in the text, the circular ablations were “drawn” as close as possible to the initia (I3-I1). Under these conditions, most of the ablations were just outside or marginally touched the primordia, these did not affect leaf polarity, even when they partially overlapped with *pDRNL* (e.g. shown in Fig. 3D, E mentioned on page 6 line 205-206).

Others went through the initia and led to growth arrest (Fig. 3A-B and S4). We have now completely separated these two types of ablations in the text to avoid confusion (explained on page 6 line 189-192). In Fig. S5 we clarify the cutting positions for I3, I1, P1 and P2 in the meristem shown in Fig. 3.

We also produce a new Figure 9 where we qualitatively and schematically summarize the outcomes of all ablations.

4. *The same point applies for the lateral ablations at leaf primordia and the incisions at sepal primordia. More information is needed on the position of these incisions in relation to what would be the fate of the cells.*

To clarify the cell fate, we now add one example of a I3 leaf initium showing the formation of lateral organ boundaries relative to *pDRNL* expression domain (Fig. S2). We also add extra diagrams to show the detailed lateral ablation positions in Fig. 7 and 8. As mentioned, we have also added a diagram summarizing the different ablation positions and outcomes (Fig. 9).

5. *Likewise, more careful descriptions are needed to interpret the outcomes of the manipulations. For example, in figure 3C, a comparison to primordia on intact meristems is absolutely critical. In addition, expression information from FIL and PRS is needed to demonstrate more convincingly that ad-abaxial polarity is unperturbed. Particularly so in the youngest primordia.*

The unwounded controls for leaf samples are shown in Fig.1 C-H and the new Figure 7E-E’’. As requested by the reviewer, we now add one extra intact, unperturbed meristem to show the flattening of leaves (I3 to P2) during 5 days (Fig.S6A-B).

© 2021. Published by The Company of Biologists under the terms of the Creative Commons Attribution License (https://creativecommons.org/licenses/by/4.0/).

As for the integration of polarity markers, we found that trichomes are always formed adaxially as in unperturbed wild type plants. To facilitate comparison, we now also show side by side transverse sections through primordia showing the expression pattern of *FIL* with or without ablation in Figure S6C (cf also Fig 1D-E, Fig.6 and 7).

6. *However, the most critical point is that for quite a percentage of primordia the effects of the manipulations are not described. In this first surgical experiment, what happens in the remaining 8/12 I3 primordia, 4/8 I2 primordia, and 2/12 I1 primordia? In this and the subsequent experiments quite some primordia are reported to arrest growth. The basis for this is not addressed.*

We agree that this was a bit hidden in the text. In the first experiment (12 radial ablations) there were two categories: (i) 22 I3-I1 initia were not significantly affected, (ii) 12 I3-I1 initia stopped growing because the ablations hit the abaxial part of the *DRNL* domain. The remaining 2 were damaged during further dissections and were not followed. We initially mixed these cases in the text which obviously led to confusion. We have now more clearly separated these two categories of ablations (page 6 for intact, page 7 for partially ablated primordia, see also summary Fig. 9). Two primordia that were damaged during dissection were not considered.

7. *This point is particularly relevant as growth seems to arrest with a few days delay from the manipulations (e.g. Figs. 4B, 6C, 7A, 7B) and expression of FIL and DRN is lost. What follows what?*

First expression is lost, then growth stops in these cases. The small outgrowths can be interpreted as wound effects, small bumps that should not be considered as aborted primordia as they don't express *FIL* or *DRNL* (Fig. 6B-B''; Fig.7A' and B'). This is equivalent to the circular outgrowths after circular ablations (Fig. S4). This is now explained on page 8 line 259-262, line 269-273 and page 11 line 379-384.

8. *Thinking about the surgical experiments shown in Fig. 6 and the statement on page 7, that some leaf primordia with limited growth show extended FIL expression, it needs to be addressed whether loss of ad-abaxial polarity underlies the growth arrest, or whether the reverse is the case. Again, at least for the surgical experiments in tomato, only a subset of surgically-isolated primordia showed a polarity defect.*

After certain lateral ablations, the *FIL* expression domain is not completely lost, but reduced in width. Growth of these primordia is very slow, although *FIL* remains abaxial. Much later on, *FIL* extends to the adaxial side (Fig 7C-C"). Therefore, the loss of polarity does not underlie reduced growth, but we cannot exclude that the reduced growth causes a change in polarity. This is explicitly mentioned on p8 line 273-278.

9. *Related to this, ablation of the adaxial side of primordia reorients growth leading to establishment of a new meristem-organ boundary below the wound (page 6). Can it be excluded that this new boundary be responsible for ad-abaxial polarity in the subtending primordium?*

No, this cannot be excluded, and this is an interesting point that we now briefly discuss (p 10 line 360-367).

10. *Figure 4D also shows additional growth between the incision and the primordium below. How might this contribute to the lack of phenotypes observed?*

We think the reviewer refers to figure 3D? As the reviewer suggested in the previous question, local events (e.g. the formation of a new boundary or extra cell growth induced by the wound) might very well be involved in repatterning the primordium after outgrowth.
This is mentioned in the discussion (p 10 line 360-367)

© 2021. Published by The Company of Biologists under the terms of the Creative Commons Attribution License (https://creativecommons.org/licenses/by/4.0/).

In short, a more comprehensive careful analysis/description of the manipulations and the phenotypes that follow is needed to allow a full assessment of effects on ad-abaxial polarity.

We agree and hope that we have addressed this request in a satisfactory manner.

Additional points:

11. For figure 7C, it is stated that polarity is not affected up to 6 DAA (although it is subsequently). The first is not obvious from the data shown. Transverse FIL sections compared to normal primordia would be informative. As mentioned above, such data and comparisons to normal primordia are also needed in other figures.

We thank the reviewer for pointing out this issue, as this was indeed not fully clear. We now include an unwounded control in Figure 7E-E" and sections showing *pFIL* expression in Fig 7D-D". We have indicated more clearly the different limits between the boundary, adaxial and abaxial domains (Fig. 7C" and D'').

12. The labeling in Fig 5 is not entirely legible.

The labeling is now improved.

13. Please include also a top view of the incisions reported in Figure 6. Here it should be noted that Sussex reports that smaller incisions at primordia that fail to fully isolate it from the rest of the meristem do not perturb dorsiventrality.

This has been added to Figure 6A' and B'. The circular ablations are of course large enough and we mention in the text that the smaller incisions are only used to eliminate specific parts of the initia/primordia and are sufficiently large for this purpose (p7 line 251-253).

Reviewer 3 Comments for the Author:

It might be that different choices of markers genes used to guide ablation or monitor consequences thereof would have yielded different results. However, these types of experiments are non-trivial and only so much can be done for one study so I think the appropriate path is to rethink the data under this perspective and when needed recast interpretations given the points below.

We thank this reviewer for his/her understanding.

1. At inception FIL has been shown to express in both the abaxial (away from the shoot axis) and adaxial regions of the primordium at inception (Caggiano et al 2017, Tamashige et al 2013). Also, FIL doesn't show expression adaxial to P1, coinciding with the zone of PIN1 polarity reversal at P1 stage (also seen in this study and in agreement with prior work). FIL becomes more abaxial when the leaf primordium has grown out of the meristem so at quite a late stage. Work from Y. Eshed J Bowman has indicated that Yabby's are likely predominantly involved in growth rather than tissue polarity (Eshed et al Development 2004 131:2997-3006; doi: 10.1242/dev.01186) Also please see Goldschidt et al Plant Cell 2008 May;20(5):1217-30. doi: 10.1105/tpc.107.057877. Epub 2008 May 9 on YABBY related signalling that merits more discussion here. The conclusion from all this is that FIL may not be a typical abaxial marker like KANADI and that more specific conclusions are needed that incorporate more nuanced views on how FIL functions.

Thanks for pointing this out. We are aware of the dynamic expression of *pFIL* during leaf initiation. Indeed, *pFIL* initially expressed overlapping with *pDRNL* and we now clearly specify this point by saying: "They (*pFIL* and *pPRS*) largely overlap with the *pDRNL* maximum, and no apparent

© 2021. Published by The Company of Biologists under the terms of the Creative Commons Attribution License (https://creativecommons.org/licenses/by/4.0/).

polarization of these markers is distinguishable before P1" (p4- p5 line 148-150)." And at later stages *pFIL* markes the abaxial domain and the middle domain. In the discussion, we mention that FIL may act as a growth activator. However, *FIL* can be used to monitor later polarity events and to show changes induced in local patterning after ablation (p11 line 381-385).

2a. Similarly regarding the choice of adaxial cell-type marker DRN, Figure 1D (D3) shows DRN expression in the boundary between the meristem and the initiating leaf where the dorsal marker REV would be absent and KANADI expression would re-establish, creating a new boundary for the next primordium to initiate. This point needs discussion.

We find indeed that *DRNL* is expressed in the future boundary (now specifically mentioned on p4 and p5), but before *KAN1* would be induced, i.e. before a physical boundary is formed. In fact, comparing our results with those reported by Caggiano et al, *DRNL* and *REV* show a very similar behaviour, and *REV* is very likely at least weakly expressed in the future boundary as well. There is also evidence that both factors interact. *KAN1* has a more complementary pattern. The relative expression dynamics are now discussed on page 10 line 341-349.

2b. Related In the same figure, stage at day 5 shows pDRN expression mainly in the pro- vasculature and subepidermal area as well as an absence from the dorsal cells. This doesn't quite match with data shown in Figure S1. What do the authors think on this? If this cannot be resolved with more data or more careful analysis of staging in existing data then maybe more replicates/more detailed substance could help address this variation in the future and this point can be clarified in discussion.

We agree that this might be confusing. This is related to the fact that the P2-P8 stages are not very clearly defined in the literature as they correspond to the number of visible primordia on a particular meristem. Thus P6 on one meristem might correspond to P8 on another one. What both figure 1D and S1 show, is that around P6 *DRNL* is still mostly adaxial, while slightly later, it retracts to the subepidermal area and the pro-vasculature. In particular, a better staging method is required, and *DRNL* as well as the other markers might help to do that. We prefer, however, to leave that discussion for another article. We have tried to clarify this in the result section (p6 line 185-189).

3. This data set as well as previous ones using ablations lack precise tracing of dorsal ventral cell lineages using dorso-ventral markers to really explain the outcome for organ polarity. Given their set-up (bigger meristems) and ability to monitor ablated primordia over time this would be possible here. This path could be highlighted in the discussion as a future step for the field.

Thank you for this suggestion. We add the point in the first paragraph of the discussion (p9 Line 309-313).

The following related points pertain to specific interpretations provided.

4. Line 122- No apparent polarity is distinguishable before P1 when the primordia start to bulge out.

According to data based on live imaging, polarity is clear since the onset of the leaf primordium inception: There may be some cell-level fluctuations (Yu et al 2017) but overall, the dorsoventral boundary appears to remain robust, unless perturbed by changes in auxin levels (Caggiano et al 2017). Please discuss accordingly.

We now modified the description of the sentence as "No apparent polarization of these markers is distinguishable before P1" (p4-p5 line 148-150).

© 2021. Published by The Company of Biologists under the terms of the Creative Commons Attribution License (https://creativecommons.org/licenses/by/4.0/).

5. Line172 ablations were performed at I3, when pDRNL was only just becoming upregulated and pFIL was not expressed at all, leaf polarity was not perturbed (Fig 3E). Given the REV is established earlier than DRN, REV, KAN and PIN1 markers would have been important when trying to estimate the relative stages of primordia and the establishment of the ad/ab boundaries. The current images do not show if the wound is already passing through the KAN expression domain surrounding the primordium (for example this would be the case in P1 and P2 where DV boundaries should have stabilized by this stage). Please discuss accordingly.

We show that *DRNL* is, like *REV*, expressed at low levels in the meristem centre (but in internal layers only). Both are strongly upregulated when organ initiation starts. We therefore now conclude that *REV* and *DRNL* have similar expression dynamics (p10 line 341- 349), At I2/I3 both *REV* and *DRNL* very likely overlap and mark the future adaxial domain.
Therefore, the ablations at I2 and I3 shown in Fig. 3A do probably not touch the KAN1 domain (KAN1 will be activated later). Future work should indeed be extended to other markers of polarity and we have discussed this more in detail now (p10 line 341-349 and p11 last paragraph).

6. The tangential ablations to disrupt adaxial and abaxial sides of the primordia are very good. However, since FIL shows expression in adaxial cells at very early stages, in this experiment, it may be that not all adaxial cells were ablated. Wound-induced KAN expression adaxial to some of the left over adaxial cells could have led to re-estabishment of the ad-ab boundary. The lineages of the remaining adaxial cells may have been sufficient to grow further and still maintain the ad-ab polarity of the ablated organ. That may explain the absence of polarity defects in the regenerating primordium. Also, it seems that the wound is not deep enough which may have contributed to the effects seen. Please discuss.

It is difficult to judge if sufficient adaxial cells would be left after the ablations to harbour both a new ab-adaxial boundary and ectopic KAN1 expression. However, we agree that other scenarios are possible. We now discuss such a scenario which proposes a dedifferentiation process followed by local repatterning (page 10 line 360-367) which is more or less what the reviewer implies. With regard to the wounding, we have determined that the wounds are about 2-3 cells deep (see also reply to reviewer 1). Although we know that this can substantially perturb local growth and e.g. auxin transport, we do not know till what extent the full patterning process is perturbed.

7. Similar considerations apply to the ablations performed in floral meristem as shown in Figure 8. One curious thing in Fig 8C, is that it seems there is ectopic PRS expression upon wounding. PRS expression is promoted by auxin and regions surrounding wounds have been shown to be associated with low auxin levels. What then explains ectopic PRS expression there? Is that also seen in the vegetative meristems?

Yes, this is an interesting phenomenon. It also seems to us that the wounding could somehow induce the expression of *PRS*. We looked at ablations performed on vegetative and inflorescence meristems. However, we have not yet obtained a convincing result showing the induction of *PRS* by wounding. At this stage, we prefer to leave it as an interesting direction for the next step to explore.

Minor point

8. Please define plastochron the first time it is used: Readers not familiar with shoot development or plant biology will probably not know the term plastrochrones is I think French I suggest using the English "plastrochrons"

We now use plastochron(s) and define it properly (p4 line 117-118).

© 2021. Published by The Company of Biologists under the terms of the Creative Commons Attribution License (https://creativecommons.org/licenses/by/4.0/).

Second decision letter

MS ID#: DEVELOP/2020/198820

MS TITLE: Stable establishment of organ polarity several plastochrons before primordium outgrowth in Arabidopsis

AUTHORS: Feng Zhao and Jan Traas

I have now received all the referees reports on the above manuscript, and have reached a decision. The referees' comments are appended below, or you can access them online: please go to BenchPress and click on the 'Manuscripts with Decisions' queue in the Author Area.

The overall evaluation is positive and we would like to publish a revised manuscript in Development, provided that the referees' comments can be satisfactorily addressed. Please attend to all of the reviewers' comments in your revised manuscript and detail them in your point-by-point response. If you do not agree with any of their criticisms or suggestions explain clearly why this is so.

We are aware that you may currently be unable to access the lab to undertake experimental revisions. If it would be helpful, we encourage you to contact us to discuss your revision in greater detail. Please send us a point-by-point response indicating where you are able to address concerns raised (either experimentally or by changes to the text) and where you will not be able to do so within the normal timeframe of a revision. We will then provide further guidance. Please also note that we are happy to extend revision timeframes as necessary.

Reviewer 1

Advance summary and potential significance to field

The changes the authors have made have improved and clarified the paper. We like the new organization of the text, adding in the non-ablated control next to the ablation, and the cartoon illustrations of the experiments. We appreciate the inclusion of the reference for the change of FIL expression in radialized leaves. Due to practical limitations, the authors could not fully address everything the reviewers requested; however, they have presented what they did straightforwardly, without over-interpreting the results.

Comments for the author

Minor comments:
Line 209 add a figure reference for the FIL data.

For Figure 3E please add the timing at which the phenotyping was done (i.e. 5DAA) to either the table or figure legend.

Line 218-219 “Once the primordia had already started to bulge out, lateral ablations of 3 to 8 cells wide did not affect growth and leaf polarity (13/13 of P1 and P2).” Please add a figure reference or the data in the supplement.

Reviewer 2

Advance summary and potential significance to field

In this revised manuscript, the authors have clarified a number of the ReviewerÂ’s comments. From the data, it is clear that incisions just adaxial to the DRNL expression domain at I2 (or I3, four instances) till P2 fail to trigger formation of radialised leaves. Similarly for sepal primordia. The situation in Arabidopsis thus is different from that reported previously in potato and tomato, which

© 2021. Published by The Company of Biologists under the terms of the Creative Commons Attribution License (https://creativecommons.org/licenses/by/4.0/).

is of interest to the readers of Development. Similarly, the observation that cells expressing FIL are required for organ growth is of interest to the readers of Development.

Comments for the author

However, the manuscript would benefit from another round of revision where the authors are more stringent regarding statements as to whether or not ad/ab polarity is established, stable, or unaffected. The data presented does not support the strong conclusions drawn in this regard. The reported data does not resolve when ad/ab polarity is first established. The authors cannot rule out that this axis is established at I1 or even later. Indeed, FIL and PRS expression is first detected at I1, and polarization of the three markers is first seen at P1 (lines 147-150). That incisions adaxial to DRNL at I1, I2 (or I3) do not block organ flattening, would support the idea that a signal from the meristem is not required for ad/ab polarity. Any conclusions on when ad/ab polarity is first established need to be toned down.

Likewise, the authors have to be more precise with statements on how incisions impact stability of ad/ab polarity. With “polarity” the authors here seem to refer to primordium flattening and the occurrence of at least some adaxial trichomes. However, young primordia of mutants such as kan1 kan2 are flattened to a degree, even though ad/ab polarity is clearly perturbed. Without marker data, it is impossible to conclude whether the ad/ab boundary is stable or correctly positioned following incision, as the authors conclude (lines 394-395). In fact, looking at Figs 3B, 3C, and S4, the primordia by no means look normal. Also, Figs 1A and 3A show DRNL expression normally persists in primordia until at least p8 or p9. Yet, in Fig 3B, DRNL expression is lost. What does DRNL expression look like in the primordia shown in Fig 3C? Therefore, the strong statement (Lines 220-221) “lateral ablations at the boundaries and ablations between the vegetative meristem and initiating organs did not perturb polarity from I3 onwards” needs revision.

Even more so, lateral ablations to 4-6 cells were performed at I2 (2x) and I1 (5x), not I3. The I2 primordia at 3DAA is no longer position between the incisions, but is displaced further down, suggesting that local patterning is perturbed but restored afterwards. Following the lateral ablations to 3-4 cells shown in Fig. 7C’, FIL expression becomes focuses on the central region of the primordium only (see 6DAA). Transverse sections through these and normal primordia are needed to support the conclusion that there are no changes in gene expression associated with polarity. Also adaxial ablations, performed only at I1 and P1 (n=5 total), lead to a loss of FIL expression and a reestablishment of polarity only several days later. The statement “At I1 or P1 no change in the 'final' leaf polarity was observed (lines 253-254) while formally true, does not really capture the situation. Ablations on the abaxial side lead to growth arrest. As per the response to Reviewers, “first FIL and DRNL expression is lost, then growth stops in these cases”.

Statements such as on lines 315-317 “We find that organ polarity is determined in a stable manner in the vegetative meristem at least two or three plastochrons before the organs grow out, at a moment when pDRNL is upregulated and organ founder cells are selected.” and lines 395-397 “Indeed, we find that in Arabidopsis the intrinsic information contained within the primordium is able to withstand major perturbations in local shape and signaling, long before polarity is fully resolved.” are not supported by the data presented. In fact, major perturbations in growth and gene expression are observed. However, in certain instances, the meristem recovers, new growth is initiated and gene expression is in part reestablished. The authors mention this but then ignore this in the final conclusions.

Similar statements in the abstract and the end of the introduction also need revision.
See also the author’s response to reviewers. Quote:
So, as Caggiano et al, we find that ablation perturbs polarity gene expression, but where they find the local activation of the abaxial marker KAN1, we find the inactivation of the abaxial marker FIL (Fig. 6A’-A’’). In addition, our result also shows that these ablations do not permanently wipe out local polarity, in contrast to what might happen in tomato or potato. This would overall suggest that local patterning is only transiently perturbed and restored afterwards.

© 2021. Published by The Company of Biologists under the terms of the Creative Commons Attribution License (https://creativecommons.org/licenses/by/4.0/).

Additional points:
The authors generated circular incisions on 12 vegetative meristems. The position of these incisions has to an extend been clarified in the revised manuscript. The circular incisions at I3 are often below or though the DRNL expression domain (Fig. S5). Likewise for some I2 and I1 primordia (lines 259-261). Please be as precise as possible. Do incisions pass through the DRNL domain, or are they below the DRNL expressing cells?

Lines 379-385: "Although we could not induce a clear change in polarity, a number of lateral ablations clearly comprised outgrowth of leaves. This was correlated with the reduction of the FIL expression domain in width and even a loss of pFIL signals in cases where very few cells were left (Fig.7A-A'). This is in line with the fact that FIL and other YABBY genes are proposed as growth activators (Eshed et al., 383 2004; Goldshmidt et al., 2008). However, FIL can be used to monitor later polarity events and to show changes induced in local patterning after ablations (Fig. S6C)."

The authors seem to contradict themselves here. If FIL is a marker for polarity, loss or changes in expression would indicate a change in polarity. Perhaps not a complete loss of polarity, but partial defects in polarity. For sure, polarity is not stable.

Summary tables like in Fig. 3E are really helpful.

Reviewer 3

Advance summary and potential significance to field

They use laser ablation of selected areas in initiating primordia (e.g. adaxial or abaxial) to determine effects on tissue polarity. These experiments can help inform on the timing of polarity establishment and also can help understand whether signals travel between the shoot apical meristem and lateral organ primordia and vice versa.

Comments for the author

I think the authors have engaged with the reviews constructively and revised accordingly to provide balanced interpretations when needed.

Second revision

Author response to reviewers' comments

Reviewer 1 Comments for the Author:

Minor comments:

1. *Line 209 add a figure reference for the FIL data.*

This has been done (line 207).

2. *For Figure 3E please add the timing at which the phenotyping was done (i.e. 5DAA) to either the table or figure legend.*

The timing has been added in the legend (line 667).

3. *Line 218-219 "Once the primordia had already started to bulge out, lateral ablations of 3 to 8*

© 2021. Published by The Company of Biologists under the terms of the Creative Commons Attribution License (https://creativecommons.org/licenses/by/4.0/).

cells wide did not affect growth and leaf polarity (13/13 of P1 and P2)." Please add a figure reference or the data in the supplement.

We now have provided a new figure in supplementary file (new Fig.S7).

Reviewer 2 Comments for the Author:

1. However, the manuscript would benefit from another round of revision where the authors are more stringent regarding statements as to whether or not ad/ab polarity is established, stable, or unaffected. The data presented does not support the strong conclusions drawn in this regard. The reported data does not resolve when ad/ab polarity is first established. The authors cannot rule out that this axis is established at I1 or even later. Indeed, FIL and PRS expression is first detected at I1, and polarization of the three markers is first seen at P1 (lines 147-150). That incisions adaxial to DRNL at I1, I2 (or I3) do not block organ flattening, would support the idea that a signal from the meristem is not required for ad/ab polarity. Any conclusions on when ad/ab polarity is first established need to be toned down.

The reviewer is right, our data do not show at what stage polarity is established, but rather suggest that any signal emanating from the meristem is not required for organ polarity from I3 onwards and the way we presented this finding was ambiguous. We have therefore removed or modified the sentences referring to the timing of polarity establishment (summary, introduction, results and discussion; lines 40-42, 117- 118; 208-209, 315-318, 399-404)

2. Likewise, the authors have to be more precise with statements on how incisions impact stability of ad/ab polarity. With "polarity" the authors here seem to refer to primordium flattening and the occurrence of at least some adaxial trichomes. However, young primordia of mutants such as kan1 kan2 are flattened to a degree, even though ad/ab polarity is clearly perturbed. Without marker data, it is impossible to conclude whether the ad/ab boundary is stable or correctly positioned following incision, as the authors conclude (lines 394-395). In fact, looking at Figs 3B, 3C, and S4, the primordia by no means look normal. Also, Figs 1A and 3A show DRNL expression normally persists in primordia until at least p8 or p9. Yet, in Fig 3B, DRNL expression is lost. What does DRNL expression look like in the primordia shown in Fig 3C?
Therefore, the strong statement (Lines 220-221) "lateral ablations at the boundaries and ablations between the vegetative meristem and initiating organs did not perturb polarity from I3 onwards" needs revision.

It is true that in Arabidopsis almost all known so-called adaxialized mutants often form partially flattened leaves. These leaves do have other defects in addition, however. For example, the *kan1 kan2* leaves are still flat, but have protrusions on the abaxial side; in some multiple *kan* mutants there are clear trichomes on both sides (c.f. Eshed et al., 2004). None of these "partially polarized" features have been observed in the leaves after our circular ablations. We are not sure why this reviewer feels the primordia in Figs 3B, 3C and S4 do not look normal (cross sections with comparable shape, trichomes on one side).
Please also note that our conclusions are not only based on morphology, but also the expression pattern of *pDRNL* (e.g. an I2 case has been shown in Fig.3D) in vegetative/floral meristems and the *pFIL* expression in floral meristems:
- We did not find perturbed *pDRNL* expression pattern during the first days after ablation. When it comes to older primordia, even in unablated meristems, *pDRNL* expression can be variable at p8/p9, so this is not a good, late marker.
- *pFIL* expression was never adaxialised in sepals after ablations.

We do agree, however, that a more detailed analysis might be required to conclude that there are no problems at all with polarity. We therefore have modified the sentence as follows: "Within the limits of the markers that we have used, we did not find any indication that lateral ablations at the boundaries and ablations between the vegetative meristem and initiating organs perturbed polarity from I3 onwards." (lines 221-223)

© 2021. Published by The Company of Biologists under the terms of the Creative Commons Attribution License (https://creativecommons.org/licenses/by/4.0/).

In the discussion we now add that "further work is now required using additional markers to show that there are no more subtle impacts at all on ad/abaxial polarity." (lines 332-333)

3. *Even more so, lateral ablations to 4-6 cells were performed at I2 (2x) and I1 (5x), not I3. The I2 primordia at 3DAA is no longer position between the incisions, but is displaced further down, suggesting that local patterning is perturbed but restored afterwards. Following the lateral ablations to 3-4 cells shown in Fig. 7C', FIL expression becomes focuses on the central region of the primordium only (see 6DAA). Transverse sections through these and normal primordia are needed to support the conclusion that there are no changes in gene expression associated with polarity. Also adaxial ablations, performed only at I1 and P1 (n=5 total), lead to a loss of FIL expression and a reestablishment of polarity only several days later. The statement "At I1 or P1 no change in the 'final' leaf polarity was observed (lines 253-254) while formally true, does not really capture the situation. Ablations on the abaxial side lead to growth arrest. As per the response to Reviewers, "first FIL and DRNL expression is lost, then growth stops in these cases".*

We agree that we cannot exclude transient perturbations after circular ablations next to the initia/primordia. This has now been explicitly mentioned (line 207-209). With regard to the ablations within the primordium we agree that there has been a transient perturbation (which was mentioned before). To avoid any ambiguity, we now state: 'After ablation at I1 or P1 polarised leaves were formed (n=5, Fig. 6A-A''). This was correlated, however with an initial inactivation of *pFIL*...towards the meristem periphery, and adaxial cells regenerated from the abaxial side of the wound (Fig 6A-A'').' (lines 253-256)

4. *Statements such as on lines 315-317 "We find that organ polarity is determined in a stable manner in the vegetative meristem at least two or three plastochrons before the organs grow out, at a moment when pDRNL is upregulated and organ founder cells are selected." and lines 395-397*

This also corresponds to point 1 above and has been changed.

"Indeed, we find that in Arabidopsis the intrinsic information contained within the primordium is able to withstand major perturbations in local shape and signaling, long before polarity is fully resolved." are not supported by the data presented. In fact, major perturbations in growth and gene expression are observed. However, in certain instances, the meristem recovers, new growth is initiated and gene expression is in part reestablished. The authors mention this but then ignore this in the final conclusions. Similar statements in the abstract and the end of the introduction also need revision.

'Intrinsic information' is probably not the right term, as it would suggest that exactly the same cells will generate a new primordium, which is not necessarily the case. This was, therefore, changed to 'local information', indicating that a shift to neighbouring cells might occur (lines 40, 324-325, 399).

See also the author's response to reviewers. Quote:
So, as Caggiano et al, we find that ablation perturbs polarity gene expression, but where they find the local activation of the abaxial marker KAN1, we find the inactivation of the abaxial marker FIL (Fig. 6A'- A''). In addition, our result also shows that these ablations do not permanently wipe out local polarity, in contrast to what might happen in tomato or potato. This would overall suggest that local patterning is only transiently perturbed and restored afterwards.

Additional points:
5. *The authors generated circular incisions on 12 vegetative meristems. The position of these incisions has to an extend been clarified in the revised manuscript. The circular incisions at I3 are often below or though the DRNL expression domain (Fig. S5). Likewise for some I2 and I1 primordia (lines 259-261). Please be as precise as possible. Do incisions pass through the DRNL domain, or are they below the DRNL expressing cells?*

© 2021. Published by The Company of Biologists under the terms of the Creative Commons Attribution License (https://creativecommons.org/licenses/by/4.0/).

A number of circular ablations we studied went through the *DRNL* domain of the initia (example I3 in Fig. 3, Fig S5). The other were between the meristem centre and the *DRNL* domain (Fig 3). None of the ablations were below the initia. In figure S5, we have indicated this more clearly with arrows. Note that ablations going through the abaxial part of the DRNL domain stopped growing.

6. Lines 379-385: "Although we could not induce a clear change in polarity, a number of lateral ablations clearly comprised outgrowth of leaves. This was correlated with the reduction of the FIL expression domain in width and even a loss of pFIL signals in cases where very few cells were left (Fig.7A-A'). This is in line with the fact that FIL and other YABBY genes are proposed as growth activators (Eshed et al., 383 2004; Goldshmidt et al., 2008). However, FIL can be used to monitor later polarity events and to show changes induced in local patterning after ablations (Fig. S6C)." The authors seem to contradict themselves here. If FIL is a marker for polarity, loss or changes in expression would indicate a change in polarity.

Perhaps not a complete loss of polarity, but partial defects in polarity. For sure, polarity is not stable.

We agree. Polarity was lost in some of the non-growing organs, while *FIL* could also be adaxial in some of the non-growing bumps. We have rephrased this and argue that these changes in polarity might be linked to growth arrest (and hence to indirect effects), but also that cause and effect are unclear at this stage (lines 381-390).

Summary tables like in Fig. 3E are really helpful.

Reviewer 3 Comments for the Author:
I think the authors have engaged with the reviews constructively and revised accordingly to provide balanced interpretations when needed.

We thank the endorsement of the reviewer.

Third decision letter

MS ID#: DEVELOP/2020/198820

MS TITLE: Stable establishment of organ polarity several plastochrons before primordium outgrowth in Arabidopsis

AUTHORS: Feng Zhao and Jan Traas

ARTICLE TYPE: Research Article

I am happy to tell you that your manuscript has been accepted for publication in Development, pending our standard ethics checks.

© 2021. Published by The Company of Biologists under the terms of the Creative Commons Attribution License (https://creativecommons.org/licenses/by/4.0/).
